# Functional analysis of sense organ specification in the *Tribolium castaneum* larva reveals divergent mechanisms in insects

**DOI:** 10.1186/s12915-021-00948-y

**Published:** 2021-02-05

**Authors:** Marleen Klann, Magdalena Ines Schacht, Matthew Alan Benton, Angelika Stollewerk

**Affiliations:** 1 School of Biological and Chemical Sciences, Queen Mary University of London, Mile End Road, London, E1 4NS UK; 2 Marine Eco-Evo-Devo Unit, Okinawa Institute for Science and Technology (OIST), 1919-1 Tancha, Onna-son, Okinawa, 904-0495 Japan; 3 Department of Zoology, University of Cambridge, Downing St, Cambridge, CB2 3EJ UK

**Keywords:** *Tribolium castaneum*, Sense organ development, Sense organ subtypes, Evolution, Gene expression, RNA interference

## Abstract

**Abstract:**

Insects and other arthropods utilise external sensory structures for mechanosensory, olfactory, and gustatory reception. These sense organs have characteristic shapes related to their function, and in many cases are distributed in a fixed pattern so that they are identifiable individually. In *Drosophila melanogaster*, the identity of sense organs is regulated by specific combinations of transcription factors. In other arthropods, however, sense organ subtypes cannot be linked to the same code of gene expression. This raises the questions of how sense organ diversity has evolved and whether the principles underlying subtype identity in *D. melanogaster* are representative of other insects. Here, we provide evidence that such principles cannot be generalised, and suggest that sensory organ diversification followed the recruitment of sensory genes to distinct sensory organ specification mechanism.

**Results:**

We analysed sense organ development in a nondipteran insect, the flour beetle *Tribolium castaneum*, by gene expression and RNA interference studies*.* We show that in contrast to *D. melanogaster*, *T. castaneum* sense organs cannot be categorised based on the expression or their requirement for individual or combinations of conserved sense organ transcription factors such as *cut* and *pox neuro*, or members of the Achaete-Scute (*Tc ASH*, *Tc asense*), Atonal (*Tc atonal*, *Tc cato*, *Tc amos*), and neurogenin families (*Tc tap*). Rather, our observations support an evolutionary scenario whereby these sensory genes are required for the specification of sense organ precursors and the development and differentiation of sensory cell types in diverse external sensilla which do not fall into specific morphological and functional classes.

**Conclusions:**

Based on our findings and past research, we present an evolutionary scenario suggesting that sense organ subtype identity has evolved by recruitment of a flexible sensory gene network to the different sense organ specification processes. A dominant role of these genes in subtype identity has evolved as a secondary effect of the function of these genes in individual or subsets of sense organs, probably modulated by positional cues.

**Supplementary Information:**

The online version contains supplementary material available at 10.1186/s12915-021-00948-y.

## Background

In arthropods, external sense organs function at the interface of the environment and the organism [1–4]. Different types (and subtypes) of sense organs exist, all of which can generally be found across the arthropod body. However, some subtypes are clustered on specific appendages that are primarily used for specific behaviours, such as gustatory receptors on mouthparts or olfactory receptors on insect antennae [5–7]. External sense organs show a great variety of habitat- and behaviour-adapted forms and functions, ranging from the simple mechanosensory bristles of flies to the complex cuticular structures of crustacean feeding setae [8, 9]. This diversity raises the question of how the different shapes and functions have emerged in arthropods and which molecular mechanisms have facilitated their evolution.

Although there is no uniform classification of sense organs in arthropods, external and internal sense organs are generally distinguished from one another [8, 10, 11]. In our present study, we focus on external sense organs in insects. There are at least five morphological categories of external sense organs described across insect species: chaetoid, trichoid, basiconic, campaniform, and coeloconic sensilla [8, 10, 12, 13]. This implies that they have been present in the last common ancestor of insects and that sensilla falling into the same category are therefore homologous in insects. Within the categories, sensilla can be further subdivided and assigned functions based on additional characteristics (Table 1). For example, aporous trichoid sensilla are mechanosensory organs, while multiporous trichoid sensilla function as olfactory receptors [10].

**Table 1:** Categories of external sensilla of insects

Sense organ	Structure	Additional characteristics	Function
**Chaetoid sensillum**	- Bristle-like - Movably - Articulated in wide socket	- Uniporous (pore at tip) - Steep angle	Contact chemosensory organ [16]
		- Aporous - Flat angle	Mechanosensory organ [15]
**Trichoid sensillum**	- Hair-like - Articulated in tight socket	- Uniporous (pore at tip) - Steep angle	Contact chemosensory organ [16]
		- Mulitporous - Curved hair	Olfactory sense organ [16]
		- Aporous	Mechanosensory organ [16]
**Basiconic sensillum**	- Pegs - Cones	- Blunt tip - Sharp or curved - Flat angle when mechanosensory	Mechano- and chemosensory organ [15, 16]
**Campaniform sensillum**	- Dome with collar	- Variations in collar size - Dome shape	Proprioception
**Coeloconic sensillum**	- Pit with cone inside		Hygro/thermo-sensory

Arthropod sense organs arise from epithelial sensory organ progenitor cells (SOPs), which give rise to 4–5 different cell types (neurons, glia, sheath cells, and cells generating the cuticular structure, e.g. hair, socket) [8, 10, 14]. In *Drosophila melanogaster*, 5 bHLH transcription factors determine which sense organ subtypes are generated by the SOPs [10]. However, the molecular subdivision is not in line with the 5 morphological categories (Table 1) [15, 16]; rather, it classifies the physiological function of sense organs. Members of the Achaete-Scute family (*achaete* (*ac*), *scute* (*sc*), and *asense* (*ase*)) determine external mechanosensory and gustatory sense organs, while Atonal family members (*atonal* (*ato*) and *absent MD neurons and olfactory sensilla* (*amos*)) specify (external) olfactory and internal mechanosensory organs [10, 17–20].

In the first step of sense organ development, these 5 bHLH transcription factors specify SOP fate (reviewed by [10]). In SOPs that give rise to external or internal mechanosensory organs, the SOP divides asymmetrically to give rise to two daughter cells (pIIa and pIIb), which are the precursors of the accessory (e.g. bristle, socket cell) and neural cells (e.g. sensory neuron, glial cell, sheath cell), respectively. The binary decision between these two fates is regulated by Notch (N) and numb (nb) (reviewed by [10]). One to two additional divisions in the precursors generate all sensory cell types. At the same time as the SOP is produced, it starts to express a set of pan-neural sensory genes (e.g. *snail* (*sna*), *prospero* (*pros*), *ase*) [10, 21–28]. In addition, transcripts of subtype-specific genes are upregulated. The transcription factor *cut* (*ct*), for example, is expressed in external mechanosensory and gustatory sensilla, while *cousin of atonal* (*cato*) is expressed in the internal mechanosensory (chordotonal) organs and olfactory sensilla; *pox-neuro* (*poxn*) and *target of pox-neuro* (*tap*) regulate chemosensory organ development [10, 29–37]*.* When these subtype identity genes are mutated, changes in subtype identity are observed. For example, mutations in *ct* lead to transformations of external mechanosensory and gustatory sensilla into chordotonal organs [38].

Taken together, the *D. melanogaster* data show that a combinatorial code of transcription factors seems to determine sense organ subtype identity. This raises the question how this code has evolved and how, or if, it is used in other arthropod taxa or even in other insect species. Thus far, only a few studies on sense organ development in arthropods other than *D. melanogaster* exist. These studies have shown that genes known to be involved in sense organ subtype specification and cell type determination within the SOP lineage in *D. melanogaster* (*ASH*, *ato*, *ase*, *sna*, *pros*, *Notch*, *Numb*) are expressed during sense organ development. However, the genes seem to perform different/additional roles in different species (e.g. [39]). For example, *ato* shows a conserved expression in chemosensory organs of the crustacean *Daphnia magna* [9], the myriapod *Glomeris marginata* [40], and the insect *D. melanogaster*. In addition, *ato* is co-expressed with *ASH* in various types of sense organs in the crustacean, including mechanosensory sensilla [9]. Does this suggest that different codes for sense organ subtype specification have evolved in insects and the closely related crustaceans? Or is the *D. melanogaster* code not even representative for insects? In order to address this question, we analyse here the expression and function of sensory genes in the development of different categories of sensilla in the red flour beetle *Tribolium castaneum*.

## Results

### Distribution of external sensilla in the first larval stage

**Fig. 1:**
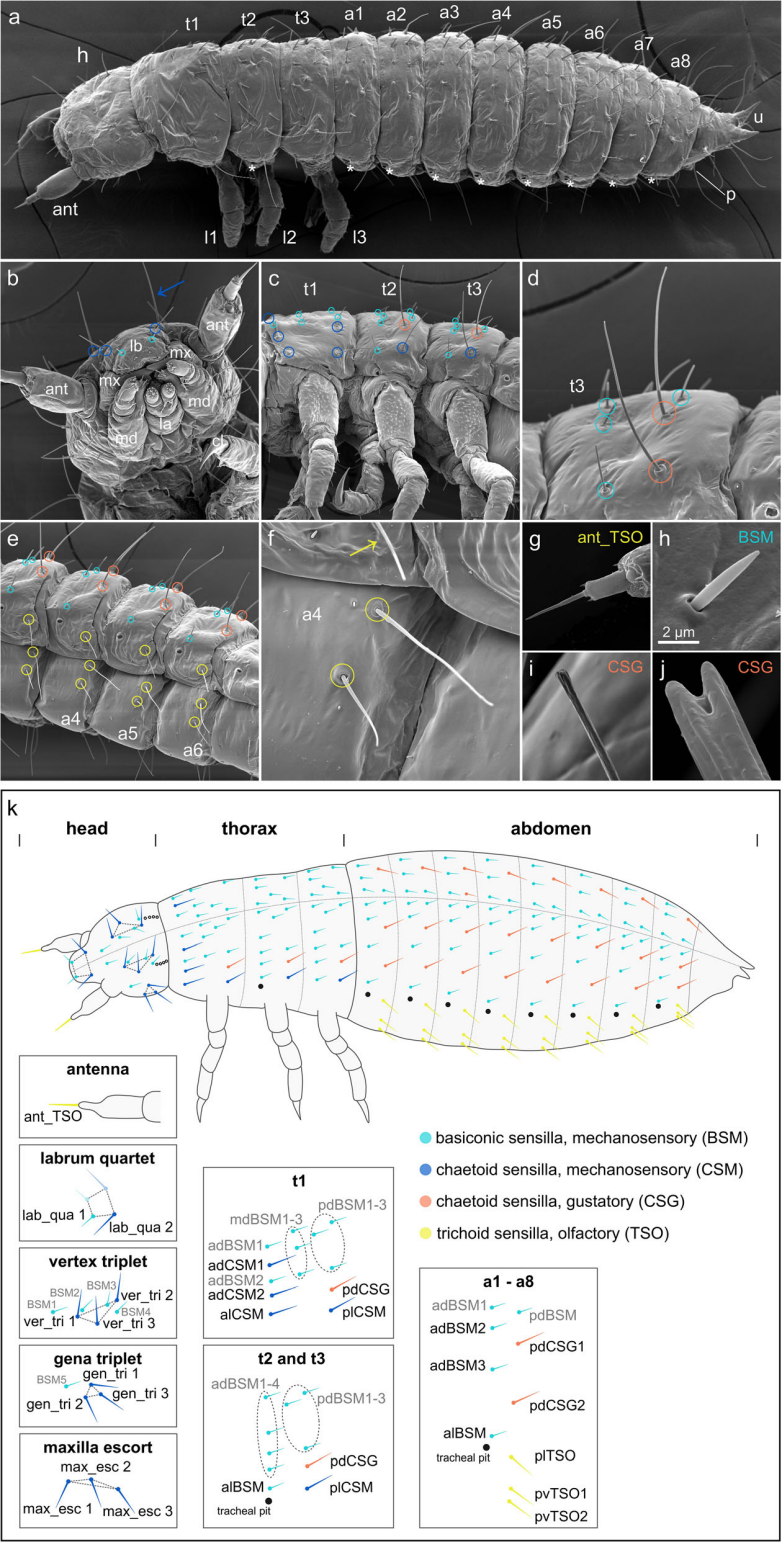
Distribution and morphology of selected external sensilla in *T. castaneum* larvae. Scanning electron micrographs (SEMs) of ESO morphology and distribution in the 1st larval stage (**a**–**i**), and morphology of sensilla in the 2nd larval stage (**j**). Anterior is towards the left in **a**–**k**. **a** Whole larva: asterisks, position of tracheal pits; ant, antenna; h, head; t1–t3, thoracic segments 1–3; l1–l3, walking legs 1–3; a1–a8, abdominal segments 1–8; u, urogomphi; p, pygopods. **b** High magnification of head (ventral view) showing mouthparts: lb, labrum; mx, maxillae; md, mandibles; la, labium; and head sensilla grouped into clusters lab_qua, ver_tri, gen_tri, max_esc [41]. **c**, **d** Thoracic segments. **e** Abdominal segments (ventro-lateral view). **f** Abdominal TSOs, **g** ant_TSO, **h** BSM. **i** In the 1st larval stage, the CSGs have a bulb-shaped tip. **j** Open pore at the tip of a CSG at 2nd larval stage. **k** Schematic representation of 1st stage larvae showing the different types of ESOs, dorso-lateral view. Grey lettering indicates sensilla, which were not analysed in the RNAi experiments. The dashed line indicates the dorsal midline. Sensilla abbreviations, head: ant_TSO, antennal trichoid sensillum (olfactory); lab_qua, labrum quartet; ver_tri, vertex triplet; gen_tri, gena triplet; max_esc, maxilla escort. Thorax: adBSM, anterior-dorsal basiconic sensillum (mechanosensory); alBSM, anterior-lateral basiconic sensillum (mechanosensory); alCSM, anterior-lateral chaetoid sensillum (mechanosensory); dCSG, dorsal chaetoid sensillum (gustatory); dCSM1–2, dorsal chaetoid sensillum (mechanosensory) 1–2; mdBSM1–3, median-dorsal basiconic sensilla (mechanosensory) 1–3; pdBSM1–3, posterior-dorsal basiconic sensilla (mechanosensory) 1–3; plCSM, posterior-lateral chaetoid sensillum (mechanosensory). Abdomen: adBSM1–3, anterior-dorsal basiconic sensillum (mechanosensory) 1–3; alBSM, anterior-lateral basiconic sensillum (mechanosensory); dCSG1–2, dorsal chaetoid sensillum (gustatory); lTSO, lateral trichoid sensillum (olfactory); pdBSM, posterior-dorsal basiconic sensillum (mechanosensory); vTSO1–2, ventral trichoid sensilla (olfactory) 1–2

In order to analyse the molecular mechanisms of sense organ development in *T. castaneum*, we first established a map of sensilla of the head and body segments of 1st instar larvae, which fell into the different morphological categories of external sensory organs (ESOs) (Table 1) and which were easily identifiable because of their prominent positions. The distribution of head sensilla has been described before [15, 41, 42], and we therefore only define here the head sensilla relevant to this study. Each antenna has one terminal trichoid olfactory sensillum (ant_TSO; Fig. 1a, b, g, k). Long mechanosensory chaetoid sensilla on the dorsal and lateral side of the head capsule are arranged in three pairs of triplets (vertex triplet (ver_tri), gena triplet (gen_tri), maxilla escort (max_esc)) on either side of the midline, and one frontal quartet (labrum quartet (lab_qua)) (Fig. 1a, b, k [41];).

In the thoracic segments, the distribution of sensilla is similar in thoracic segments 2 and 3 (t2, t3; Fig. 1a, c, k). However, thoracic segment 1 (t1) differs from t2 and t3 as it is about twice as big (Fig. 1c) and the sensilla are arranged in three anterior-posterior rows (Fig. 1c, k, anterior, medial, posterior) rather than two. Based on the morphological characteristics described in Table 1, we identified 5 chaetoid sensilla on t1, four of which are mechanosensory (dorsal chatoid sensilla 1 and 2 (adCSM1 and 2), anterior and posterior lateral sensilla (alCSM and plCSM)), while the remaining dorsal chaetoid sensillum is gustatory (pdCSG) (Fig. 1a, d, i, k). The two posterior sensilla pdCSG and plCSM are located in the same relative position in t2 and t3. In addition, t1 exhibits an anterior-dorsal basiconic mechanosensory sensillum (adBSM), a median transverse row of three dorsal BSMs (mdBSM1–3), and a posterior transverse row of three dorsal BSMs (pdBSM1–3; Fig. 1a, h, k). t2 and t3 show an anterior and posterior transverse row of BSMs only (adBSM1–4, alBSM, pdBSM1–3; Fig. 1a, d, k). One of the anterior BSMs, alBSM, is located in a prominent position lateral to the tracheal pit in t2 and t3 and can also be identified in all abdominal segments (Fig. 1a, c, e, k). In t1, tracheal pits are absent, and the same relative position is occupied by alCSM.

Similar to t2 and t3, the sensilla are arranged in an anterior and posterior row in the abdominal segments. The anterior row consists of four BSMs (adBSM1–3 and alBSM). In the posterior row, a single BSM is visible (pdBSM; Fig. 1a, e, k). In addition, each abdominal segment has five chemosensory sensilla: two chaetoid sensilla (pdCSG1 and 2) and three trichoid sensilla (plTSO, pvTSO1 and 2). pdCSG1 and 2 are located at the dorsal-posterior side of each abdominal segment and develop an open pore at the tip in the second larval stage (Fig. 1a, e, j, k), which is typical for contact chemoreceptors. One of the three trichoid sensilla is positioned at the posterior-lateral side of the abdominal segments (plTSO), at the same vertical line and posterior to the tracheal pit (Fig. 1a, e, f, k). The remaining two trichoid sensilla (pvTSO1 and 2) are located on the ventral side of the abdominal segments. The steep insertion angle and curved shape of the TSOs suggest an olfactory function.

### Expression of *Tc ASH* and *Tc ato* in the developing sense organs

**Fig. 2:**
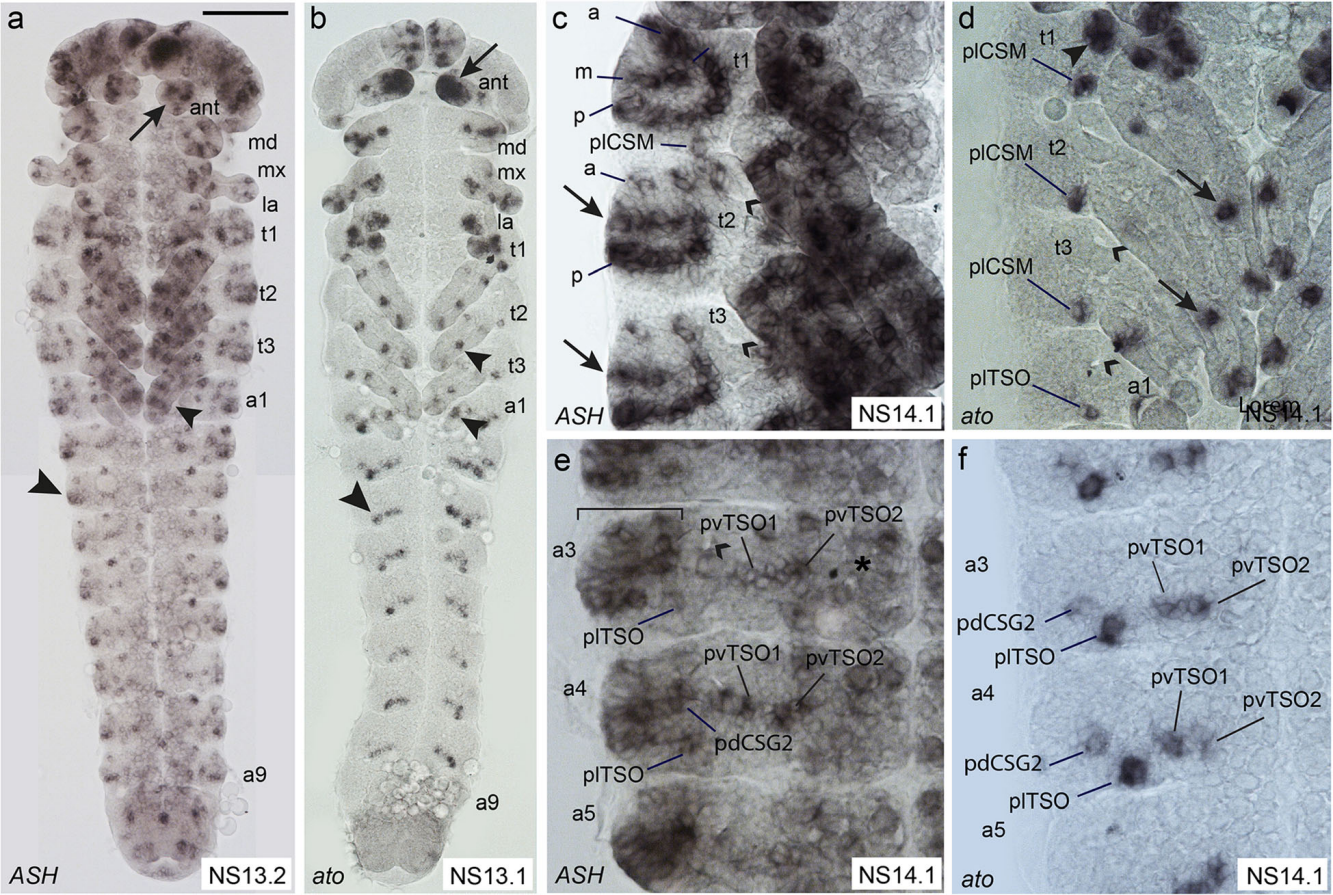
Comparison of *Tc ASH* and *Tc ato* expression patterns in the developing peripheral nervous system. Light micrographs of flat preparations stained with DIG labelled RNA probes. Anterior is towards the top. Open arrowheads: tracheal pits. **a**, **b** *Tc ASH* and *Tc ato* expression pattern in whole embryos at NS13. Arrows: expression in the antennae. *Tc ato* is strongly expressed in the whole tip of the antenna, while *Tc ASH* is expressed in small groups of cells. Small arrowheads: expression in the legs. Large arrows: expression in the lateral body wall. **c** In the three thoracic segments, *Tc ASH* is expressed in three dorso-ventral rows (anterior-dorsal (a), medio-dorsal (m), and posterior-dorsal (p)) dorsal to the appendages, covering all areas of ESOs development. Arrows: medio-lateral expression domain in t2 and t3 corresponding to an area which is devoid of ESOs in 1st stage larvae. Transcripts are visible in the legs. **d** In the thorax, *Tc ato* is expressed in a single group of cells at the posterior base of the legs, corresponding to plCSM, and a few groups in the legs (arrows). At approximately the same position, the Tc ato positive plTSO cluster is visible in the abdominal segments. Arrowhead in t1: additional *Tc ato* positive cluster. **e** In the most dorsal part of the abdominal segments (bracket), *Tc ASH* is expressed in many cells, covering the area from which the pdCSGs and BSMs arise. *Tc ASH* is also expressed in the ventro-lateral areas from which the three TSOs arise and in the ventral neuroectoderm (asterisk). **f** *Tc ato* is expressed in pdCSG2 and the three TSOs in the abdominal segments. For abbreviations, see Fig. 1. Scale bar in **a**, 100 μm in **a**, **b**; 25 μm in **c**, **d**, **e**, **f**

In *D. melanogaster*, *ac-sc* and *ato* outline the areas where sense organs form and are therefore known as proneural genes. We analysed the expression patterns of the single *T. castaneum Ac-Sc* and *ato* homologues, *Tc ASH* and *Tc ato*. We use here a staging system that has been developed previously for gene expression analysis in the central nervous system [43], with additional subdivisions that enabled us to capture the dynamic expression patterns of the sensory genes (Additional file 1: Figure S1) [43–47]. In the developing peripheral nervous system, clusters of *Tc ASH* and *Tc ato* positive cells are visible in the head, thoracic, and abdominal appendages as well as in domains lateral to the thoracic appendages and lateral to the developing ventral nerve cord in the abdominal segments (Fig. 2a, b; Additional file 1: Figure S2). *Tc ASH* is also strongly expressed in the central nervous system (Fig. 2e; Additional file 1: Figure S2; see also [44]). In the areas of sense organ development, *Tc ato* expression starts as early as NS3, while *Tc ASH* expression is not visible before NS7 (Additional file 1: Figure S2). Both genes are expressed in many domains in the appendages (Fig. 2a, b; Additional file 1: Figure S2c, e, f). Here, we focus on the areas from which the sense organs depicted in the scheme in Fig. 1k arise, except for the head capsule, where gene expression cannot be related to the larval sensilla.

*Tc ASH* is expressed in more domains than *Tc ato*, in particular in regions lateral (dorsal) to the thoracic appendages and dorsal to the *Tc ato* domains in the abdominal appendages (bracket in Fig. 2e). In most cases, it is not possible to distinguish clusters of *Tc ASH* expressing cells belonging to individual sense organs in the dorsal domains because of their close proximity. *Tc ASH* expression in the lateral body wall starts with the areas from which alBSM and plCSM develop at NS7 to NS10 (Additional file 1: Figure S2b, c). This expression persists and the pattern develops into three rows of *Tc ASH* expression (Fig. 2c: anterior, medial, posterior) dorsal to the appendages in the thoracic segments covering all positions from which larval sensilla emerge (Fig. 2c; t1: pdCSM1–2, alCSM, plCSM, and all BSMs; t2 and t3: pdCSG, plCSM, and all BSMs, including alBSM). The medial row in t2 and t3 (arrows in Fig. 2c) does not exhibit external sensilla in the 1st larval stage.

*Tc ato* is initially only expressed in alBSM between NS7 and NS10, in addition to the appendages (Additional file 1: Figure S2e, f). At NS13.1, *Tc ato* is expressed in a single cluster of cells close to the posterior base of the three thoracic appendages (Fig. 2d). This position most likely corresponds to plCSM in the larva (Fig. 1k). In addition, *Tc ato* is expressed in a large cluster of cells in t1 in the same area where the tracheal pits arise in t2 and t3 (Fig. 2d). The cluster might cover the position of several sensilla (alCSM, adCSM2, adBSM2).

In the abdominal segments, the dorsal-most *Tc ASH* domain also spreads over the areas from which all described sensilla arise: adBSM1–3, alBSM, pdBSM, and pdCSG1–2 (Fig. 2e). Four clusters of *Tc ASH* positive cells are furthermore visible ventral to the dorsal domains in all abdominal segments; one is located posteriorly and in approximately the same vertical line as the tracheal pit, and three are positioned in approximately the same horizontal line posteriorly to the tracheal pit. These positions correlate with the *Tc ato* positive domains that correspond to the positions of sense organs plTSO and pdCSG2, pvTSO1 and 2 in the 1st larval stage (Fig. 2f). In addition, *Tc ato* is strongly expressed at the tip of the antennae, which correlates with the position of the trichoid olfactory antennal sensillum (ant_TSO) in the 1st larval stage (Fig. 2b; Additional file 1: Figure S2f). *Tc ASH* clusters are also visible in the antenna but not at the very tip (Fig. 2a; Additional file 1: Figure S2c).

### Expression of genes conferring sense organ subtype identity in *Drosophila: Tc ct*, *Tc cato*, *Tc tap*

In order to examine sensory organ subtype specification, we next analysed the expression of *T. castaneum* homologues of genes that specify subtypes in *D. melanogaster*. These include *Tc ct*, *Tc cato*, and *Tc tap*. Despite numerous attempts, we were not able to obtain in situ hybridisation data for *Tc amos*, the third member of the *T. castaneum* Atonal family and *poxn*, for which we have functional data (see below).

**Fig. 3:**
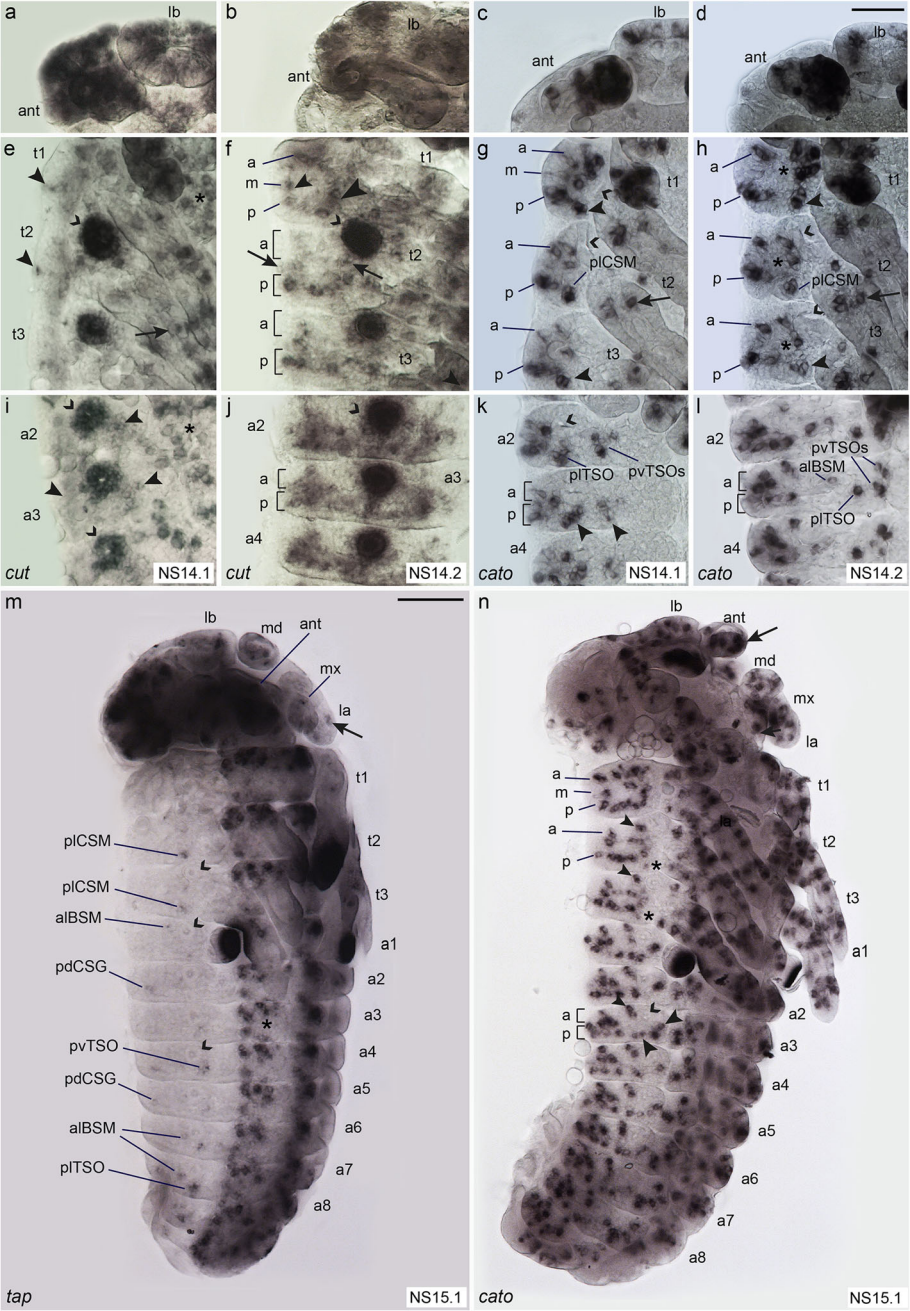
Comparison of the expression patterns of sense organ subtype-specific genes. Light micrographs of flat preparations stained with DIG labelled RNA probe of *Tc ct*, *Tc cato*, and *Tc tap*. Open arrowheads: tracheal pits; a, m, and p indicate the anterior-dorsal, medio-dorsal, and posterior-dorsal rows of expression, respectively, corresponding to the larval sensilla rows. **a**–**d** *Tc ct* and *Tc cato* are expressed in the labrum and antennae. **e** NS14.1, arrowheads: *Tc ct* expression lateral to the appendages. Arrow: leg expression; asterisks: VNE expression. **f** NS14.2, ring-like arrangement of *Tc ct*+ cells in t1; row m has two clusters, dorsal (small arrowhead) and ventral (large arrowhead). In t2–3, *Tc ct*+ cells are aligned in row p and two clusters in row m (arrows). Scattered, cells are visible in row a. **g** NS14.1: *Tc cato+* clusters arranged in a, m, and p rows in t1–3. Arrowheads: plSCM clusters. Arrow: leg expression. **h** NS14.2, *Tc cato* is expressed in rows a, p, and a medial cluster (asterisks) in t1–3. Arrowheads: plCSM. **i** NS14.1—arrowheads: expression of *Tc ct* around abdominal tracheal pits; asterisk: VNE. **j** NS14.2: *Tc ct* is expressed in row p; row a is only partially covered. **k** NS14.1, *Tc cato+* clusters are visible in rows a, p. plTSO and pvTSO1–2 have prominent positions posterior and ventral to the tracheal pits, respectively (arrowheads). **l** NS14.2: plTSO, pvTSO1–2, and alBSM indicated. **m** NS15.1, *Tc tap* is expressed in plCSMs, alBSMs, and pdCSGs. Arrows: expression in appendages. **n** NS15.1, clear arrangement of *Tc cato* positive cells in a, m, and p rows. plCSM expression is decreased (asterisks); small arrowheads: alBSM; large arrowheads: plTSO and pvTSO1–2; arrow: expression in antenna. For abbreviations, see Fig. 1. Scale bar in **d**, 25 μm in **a**–**l**; scale bar in **m**, 100 μm in **m**, **n**

*Tc ct* expression starts in the maxillary and labial appendages in NS7 and becomes visible in the antennae appendages by NS10 (Additional file 1: Figure S3a, b). The gene remains expressed in the labrum and whole antennae through subsequent stages (Fig. 3a, b). In addition, *Tc ct* is strongly expressed in ring-shaped domains in t2 to t3 and a1 to a8 from NS10 onward (Fig. 3e, f, i, j, open arrowheads; Additional file 1: Figure S3b, c). These areas develop into the tracheal pits. In the thoracic and abdominal segments, low *Tc ct* expression becomes visible dorsal to the appendages in t1 to t3 and dorsal to the developing ventral nerve cord in the abdominal segments from stage 14.1 onward (Fig. 3e, i). At this stage, expression is also present in all appendages and the CNS (Fig. 3e, i).

In the peripheral nervous system, the fully developed *Tc ct* expression pattern can be observed in NS14.2 (Fig. 3b, f, j). Similar to *Tc ASH*, *Tc ct* expression forms an incomplete ring-shape in t1, although covering a smaller region (Fig. 3f). However, in the *Tc ASH* positive area corresponding to the medio-dorsal row of setae in the larva (mdBSM1 to 3; Fig. 2c), only one *Tc ct* cluster is visible in the dorsal-most position that might correlate to expression in mdBSM1 (Fig. 3f, small arrowhead). There is no expression in the remaining part of the row, except close to the base of the first thoracic leg (Fig. 3f, large arrowhead). This area cannot be directly correlated with the position of external larval setae. In t2 and t3, *Tc ct* expressing cells are arranged in a posterior row lateral to the appendages but appear scattered towards anterior (Fig. 3f). Similar to t1, two clusters of *Tc ct* positive cells are visible in the medial area in both t2 and t3; however, these cannot be correlated with external larval setae (Fig. 3f, arrows). In the abdominal segments, the fully developed *Tc ct* expression pattern covers the complete posterior row of larval setae, including the lateral and ventral TSOs medial to the tracheal pits (Fig. 3j). The anterior and posterior expression domains form a continuous area, which takes on a triangular shape towards anterior and thus does not cover the positions of all larval setae in the anterior row (Fig. 3j).

In the peripheral nervous system, *Tc cato* is first expressed in the antennae and mandibles in stage NS7 embryos (Additional file 1: Figure S3d). In subsequent stages, additional *Tc cato* expression sites form in the antennal, maxillary, labial, and thoracic appendages (Fig. 3c, g; Additional file 1: Figure S3f). From stage NS10 onward, clusters of *Tc cato* expressing cells become visible lateral to the appendages in the thorax and lateral to the ventral neuroectoderm in the abdominal segments (Additional file 1: Figure S3f). With the appearance of additional expression domains, the clusters become arranged into rows (Fig. 3g, h, k, l, n). The developing plCSM, alBSM, plTSO, and pvTSO1 and 2 sensilla are clearly visible as separate clusters, while the remaining clusters merge into each other and are not identifiable relative to the position of the larval sensilla (Fig. 3g, h, k, l, n). The arrangement of *Tc cato* positive cells into rows is more pronounced in stage NS15.1, possibly due to the segments having narrowed along the anterior-posterior axis and extended along the dorso-ventral axis as part of germband retraction and dorsal closure (Fig. 3n). The posterior rows of *Tc cato* expression in the thoracic and abdominal segments seem to cover all larval sensilla positions, except for plCSM, where the previous expression has almost ceased. Three clusters in the medial row of t1 might correspond to the medio-dorsal BSMs1–3 (Fig. 3n). Similar to *Tc ASH* expression, a medial row of expression is also visible in t2 and t3, which cannot be correlated to external larval sensilla (Fig. 3n). In t2, t3, and the abdominal segments, there is a gap of *Tc cato* expression in the medial part of the anterior rows. In abdominal segments, however, several *Tc cato* positive cell groups cluster in the dorsal part of the anterior row, which might rearrange to cover the gap during further dorso-ventral extension of the germband (Fig. 3n). Furthermore, many *Tc cato* clusters are visible in all appendages and there is a strong expression domain at the tip of the antennae (Fig. 3n).

*Tc tap* is strongly expressed in the CNS from NS11 onward (Additional file 1: Figure S3e); however, expression is comparably low in the peripheral nervous system. It does not start before NS15.1 (Fig. 3m) and begins to decrease after about 6 h in NS15.4 (Additional file 1: Figure S3g). *Tc tap* is expressed in clusters of cells in the head appendages and in a few cells in the lateral body wall of the thorax and the abdomen (Fig. 3m). *Tc tap* positive cells are identifiable in the plCSMs, alBSMs, plTSOs, and pvTSOs as well as in one of the two abdominal pdCSGs (Fig. 3m; Additional file 1: Figure S3g).

### Expression patterns of *Tc asense*, *Tc prospero*, and *Tc snail*

**Fig. 4:**
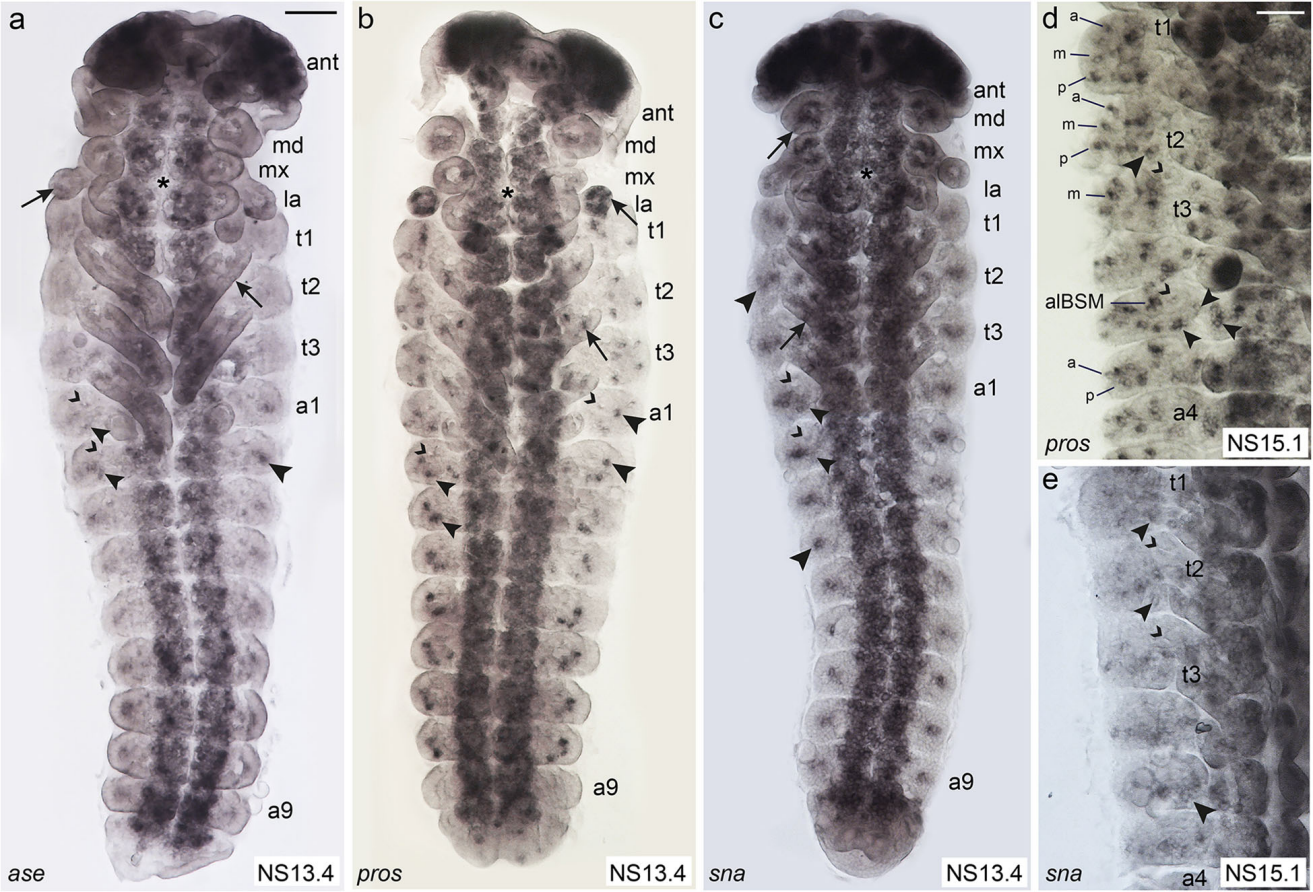
Comparison of the expression patterns of pan-neural genes. Light micrographs of flat preparations stained with DIG labelled RNA probe of *Tc ase*, *Tc pros*, and *Tc sna*. Open arrowheads: tracheal pits. **a** *Tc ase* is strongly expressed in the developing brain, the antennae, and the VNE (asterisk). Arrows: scattered *Tc ase+* cells in the remaining appendages (arrows) and the lateral body wall (large arrowhead). Small arrowheads: plTSOs. **b** Small arrowheads: *Tc pros* expression in plTSOs. Large arrowheads: *Tc pros* expression dorsal to the tracheal pits in alBSMs. *Tc pros* is also strongly expressed in the developing brain, in clusters of cells in all appendages (arrows) and the VNE (asterisk). **c** Similarly, *Tc sna* is expressed in the brain, clusters of cells in the appendages (arrows) and VNE (asterisk). Large arrowheads: *Tc sna* is expressed in large clusters in the lateral body wall. The expression extending below the tracheal pits might correspond to the developing plTSOs (small arrowheads). **d** At NS15.1, groups and single cells express *Tc pros* in the lateral body wall, which seem to cover all areas of ESO formation. Additionally, the medial row that does not give rise to ESOs expresses *Tc pros*. Due to their prominent positions relative to the tracheal pits, the alBSM clusters, the plCSM clusters (large arrowhead), and the three TSO clusters (small arrowheads; plTSO, pvTSO1–2) are easily identifiable. **e** *Tc sna* shows a transient expression pattern in groups and single cells, some of which cover the areas where sensilla appear next to landmarks, such as the plCSMs (small arrowheads) in the thoracic segments and the plTSOs in the abdominal segments (large arrowhead). For abbreviations, see Fig. 1. Scale bar in **a**, 50 μm in **a**–**c**; scale bar in **d**, 50 μm in **d**, **e**

In *D. melanogaster ase*, *pros* and *sna* are so-called pan-neural genes, which are expressed in all sense organs after SOP formation. *Tc ase* shows a strong and prolonged expression in the CNS (Fig. 4a [43, 44];); however, the expression is limited to a transient expression in a few cells and clusters in the PNS (Fig. 4a). Expression in the PNS starts at NS9 in small domains on the appendages and a few cells on each side of the lateral body wall (Additional file 1: Figure S4a). At NS11, additional clusters appear in the abdominal segments (Additional file 1: Figure S4b). One of the clusters can be identified as plTSO after formation of the tracheal pits in the abdominal segments at NS13 (Fig. 4a). The expression domain located dorsal and posterior to the tracheal pits cannot be assigned to specific sensilla (Fig. 4a). *Tc ase* expression decreases thereafter and is not detectable any more in most areas of the PNS by NS15 (Additional file 1: Figure S4f).

*Tc pros* expression is first visible in the head appendages at NS7 (Additional file 1: Figure S4c). At NS10, a single cluster of *Tc pros* positive cells appears on each side of the lateral body wall (Additional file 1: Figure S4g). By NS13.4, additional clusters are present, three of which can be attributed to the developing plCSMs (thorax) and plTSOs (abdomen) and the alBSMs (Fig. 4b). At NS15.1, *Tc pros* positive clusters seem to cover all areas of external sensilla formation (Fig. 4d). In addition, *Tc pros* is expressed in the medial area in t2 and t3 that does not give rise to external sensilla (Fig. 4d). Due to their prominent positions around the tracheal pits, alBSM, plCSM, plTSO, and pvTSO1 and 2 can be clearly identified (Fig. 4d).

*Tc sna* expression starts at NS7 in the mandibles, and by NS9, all appendages show *Tc sna* expression domains (Additional file 1: Figure S4d). Similar to *Tc pros*, bilateral *Tc snail* positive clusters appear in the lateral body wall. They appear first in the thoracic segments at NS7 and have extended to A9 by NS9 (Additional file 1: Figure S4d, e). One additional *Tc sna* expression domain is visible at NS13, which can be allocated to the abdominal plTSO due to its position posterior to the tracheal pits (Fig. 4c). During the subsequent stages, *Tc sna* shows a transient expression pattern in groups and single cells, some of which cover the areas where sensilla appear next to landmarks, such as the plCSMs in the thoracic segments (Fig. 4e). *Tc sna* expression decreases earlier than that of *Tc pros* in the PNS, although both genes continue to be strongly expressed in the CNS (Fig. 4d, e).

### *Tc ASH* and *Tc ato* have different roles in sense organ development

The different expression of *Tc ASH* and *Tc ato* in SOPs (Fig. 2) raises the possibility that these genes specify sense organ subtypes like in *D. melanogaster*. In order to test this hypothesis, we disrupted *Tc ASH* and *Tc ato* function via parental RNAi and examined sense organs in affected larvae. We focused our analysis on a subset of sensilla which were easily identifiable because of their proximity to landmarks (tracheal pits, segmental border) or arrangement relative to other sensilla. The grey lettering in Fig. 1k indicates which sensilla were not included in the RNAi study.

**Fig. 5:**
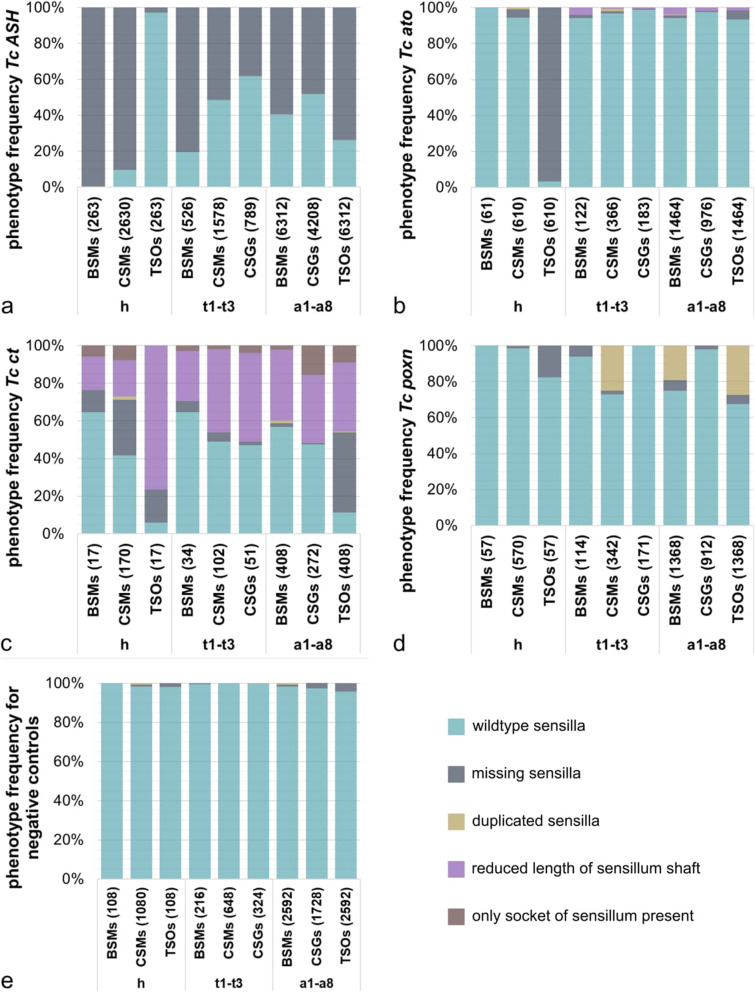
Quantification of the RNAi phenotypes of external larval sensilla. The bars represent the percentages of phenotypes identified for the different ESO subtypes (BSMs, CSMs, CSGs, and TSOs) on the head (h), the thoracic (t1–t3), and the abdominal segments (a1–a8) of *Tc ASH* (**a**), *Tc ato* (**b**), *Tc ct* (**c**), *Tc poxn* RNAi (**d**), and the negative control cuticles (**e**), respectively. Sensilla that were not affected are categorised as ‘wildtype sensilla’. Sensilla showing a phenotype are divided into the four categories ‘missing sensilla’, ‘duplicated sensilla’, ‘reduced length of sensillum shaft’, and ‘only socket of sensillum present’, if applicable. **a** We analysed 387 specimens for both non-overlapping dsRNA fragments (NOF1 and 2) of *Tc ASH* larvae in total, of which 263 showed a specific phenotype (sensilla missing). **b** We analysed 119 specimens of *Tc ato* RNAi (NOF1 and NOF2 collectively), of which 61 showed a phenotype. *Tc ato* RNAi cuticles were missing the ant_TSOs (96.72%, see 3rd bar), and also observed a small percentage of duplicated sensilla and sensilla with reduced shaft lengths. **c** We were able to analyse only 26 specimens in total for both NOFs of *Tc ct* (*n* = 17 showed a phenotype). Sensilla of *Tc ct* cuticles could be grouped into the four different categories of phenotypes. The most abundant phenotype for all ESOs types was identified as ‘sensilla with reduced shaft lengths’ (purple). **d** We performed pRNAi in *T. castaneum* pupae to examine the function of *Tc poxn*. We analysed 111 specimens in total. 51.35% of the analysed specimens showed a phenotype which were identified as duplicated sensilla (CSMs on thorax, BSMs and TSOs on abdomen). See Additional file 1: Table S4 for summary of RNAi injection results

**Fig. 6:**
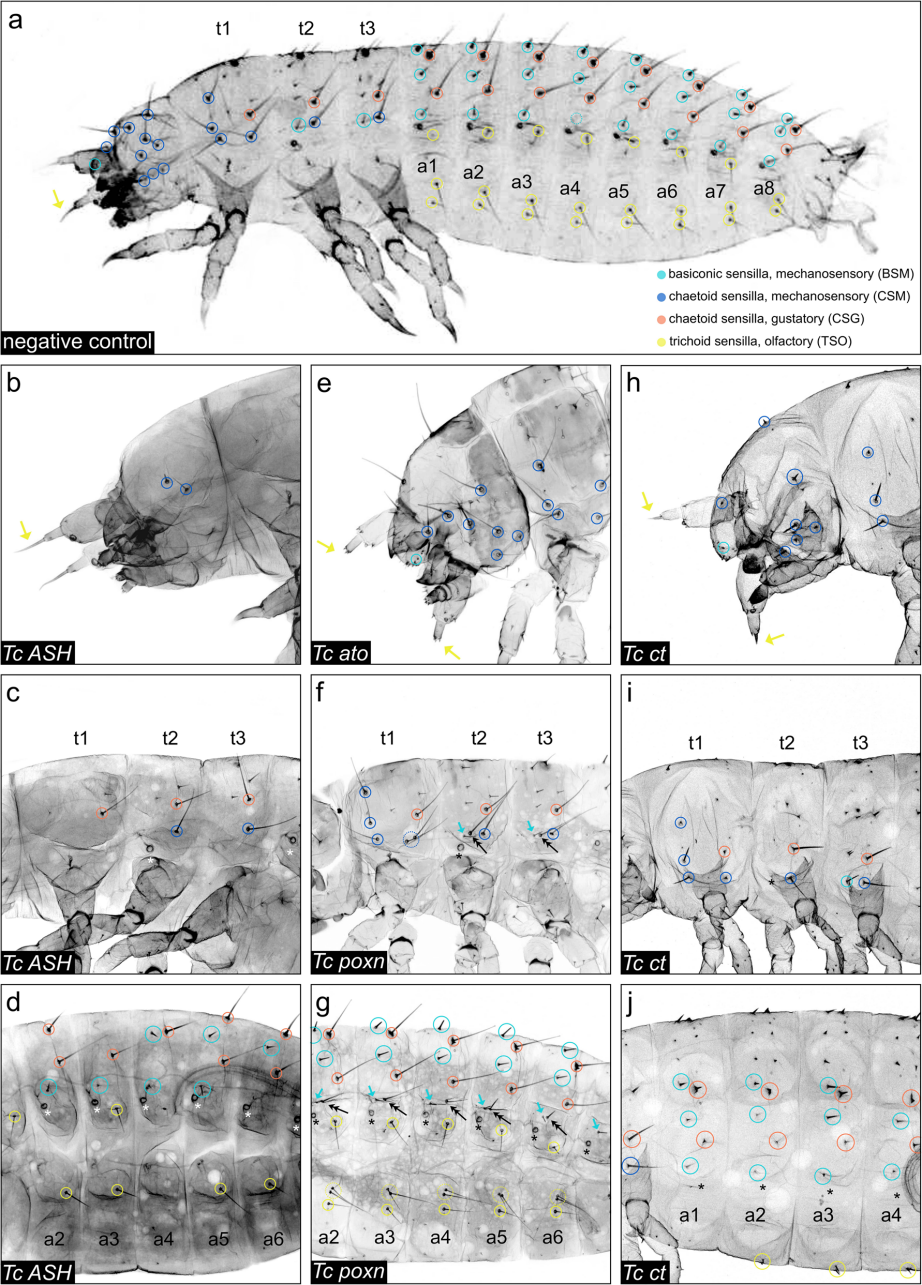
RNAi phenotypes of cuticles. Laser-scanning confocal images of L1 cuticles; anterior is to the left. **a** Sensilla analysed in RNAi experiments in a negative control cuticle. Please note that alBSM is not visible in a4 (empty turquoise circle). **b** On the head of the *Tc ASH* RNAi cuticle, only few CSGs and the ant_TSOs are present. **c** On t1–3 of *Tc ASH* RNAi cuticles, only pdCSG (t1), plCSMs and pdCSGs (t2 and t3) are present. The remaining CSMs and the two BSMs are missing. **d** On a1–8 of the *Tc ASH* RNAi cuticle, all three types of sensilla (BSMs, CSGs, and TSOs) are affected. **e** The *Tc ato* RNAi cuticle of the head shows missing ant_TSOs (yellow arrows). **f**, **g** The *Tc poxn* RNAi cuticle shows duplications of specific sensilla (plCSM, alBSM, and pvTSO1) on thorax and abdomen. **f** On the thorax, the plCSMs (dotted blue circle) are duplicated, and an additional sensillum is found between alBSM and plCSM on t2 and t3, which has the morphological characteristics of plCSMs (black arrows). **g** On a2–a8, pvTSO1s are duplicated in *Tc poxn* RNAi cuticles (dotted yellow circles). An additional sensillum is found posterior to the alBSMs in a1–6 (black arrows). The additional sensillum exhibits a longer shaft compared to the wildtype BSMs. **h** On the head of *Tc ct* RNAi cuticles, ant_TSOs (yellow arrows), CSMs and BSMs have shorter shafts (blue and turquoise circles). ver_tri1–2 are missing. **i** On t1, adCSM1, plCSM, and pdCSG develop a socket only. On t2, the alBSM is missing. **j** The shafts of the CSGs and alBSMs are shorter or only developed as sockets. plTSOs are missing; pvTSOs have shorter shafts. Asterisks in **i** and **j** indicate missing tracheal pits. Scale bar in **a**, 100 μm; scale bar in **b**, 50 μm

When examining control larvae (from parents injected with water or buffer alone), 98 to 100% of these sensilla were present (Figs. 5a and 6a; Additional file 1: Table S1). The observed variation is due to the absence of sensilla at specific positions along the anterior-posterior axis. In the head and thorax, 99.1% of the analysed sensilla are present at all positions (2461/2484), while in the abdominal segments overall 2.87% (199/6912) of sensilla are missing. The abdominal TSOs (4.32%) show the highest variability followed by the CSGs (2.60%). In order to elucidate the significance of the RNAi phenotypes, we recorded and analysed the affected sensilla separately for the head, thorax, and abdomen (Fig. 5; Additional file 1: Figure S5). For Fig. 5, we put the numbers of affected sensilla together for each sensilla category (TSOs, CSMs, BSMs, CSGs) across all larvae analysed (see Additional file 1: Tables S2-6 for summary of RNAi data).

In *Tc ASH* RNAi larvae, two types of morphologically distinct mechanosensory sensilla (basiconic and chaetoid) are missing from the head: 90.38% of the CSMs and 99.62% of the BSMs (Figs. 5a and 6b–d). In addition, 2.73% of the olfactory antennal trichoid sensillum ant_TSO are missing; however, this is only slightly above the proportion of defects seen in control larvae (1.85%; Fig. 5d; Additional file 1: Table S1). A similar pattern is seen on the thorax, where BSMs are most affected (80.42% missing) and to a lesser extent the CSMs (51.52% missing) (Fig. 6c) In addition, 38.15% of the gustatory chaetoid sensilla, the CSGs, are absent on the thorax (Fig. 5a). The intermediate phenotype in the CSMs could indicate that *Tc* ato, which is expressed in the thoracic CSMs, can partially replace the role of *Tc ASH*. Alternatively, other proneural and/or positional factors act together with ASH, which have not been analysed here. This might also explain the low effect of *Tc ASH* RNAi on the CSGs which do not co-express *Tc ato*.

Although the head TSO pattern is not significantly changed in Tc ASH RNAi larvae, the TSOs are the most affected sensilla in the abdominal segments. 73.80% of the TSOs (plTSOs, pvTSOs 1 and 2) are missing in the abdomen, followed by 59.28% of the BSMs and 48.05% of the CSGs (Figs. 5a and 6d). In the most severe *Tc ASH* RNAi phenotypes, all sensilla are missing (Additional file 1: Figure S5d, f, j). This is in line with previous *Tc ASH* RNAi data in adult beetles [48]. In addition to the sensilla phenotypes, the pretarsal segments of the legs, the urogomphi, and the mandibles appear rounded (Additional file 1: Figure S5b, h, l compare to Additional file 1: Figure S5a, g, k). The ‘rounded pretarsal segment’ phenotype has also been documented in the iBeetle RNA interference screen and in another insect [49, 50]. Furthermore, the tarsal claws are missing in *Tc ASH* RNAi adult beetles [48]. Taken together, our results clearly show that *Tc ASH* is required for the generation of all subtypes of ESOs analysed, except for the antennal TSOs.

In contrast to *Tc ASH*, *Tc ato* has only a minor role in the formation of larval ESOs. This is similar to *D. melanogaster* larvae, where *ato* is only required in a single combined olfactory/gustatory dorsal organ besides regulating the development of internal stretch receptors [17, 51]. In *T. castaneum*, the antennal TSOs are the only ESOs that are frequently missing (96.72%) in *Tc ato* RNAi (Fig. 5b, dark blue bar; Fig. 6e). All other ESOs are present on the head, and on the thorax and abdomen, BSMs, CSMs, CSGs, and TSOs are only missing to a small percentage, which is in the range of the variations seen in the controls (0.55–4.92%; Fig. 5b). The differentiation of all types of sensilla is also affected at a small rate in the thorax and abdomen: the sensilla are duplicated or have shorter shafts compared to wildtype (0.1–1.37%, beige and 0.55–4.10%, purple in Fig. 5b). While this could be due to off-target/toxic effects of the dsRNA, *Tc ato* could also function during sensilla morphogenesis at a late stage of development that was not captured in our analysis.

### Functional analysis of the ‘subtype’ identity genes

Next, we analysed the role of the *T. castaneum* homologues of genes that specify subtype identity in *D. melanogaster*, namely *Tc ct*, *Tc poxn, Tc amos*, *Tc cato*, and *Tc tap*. Parental *Tc ct* RNAi resulted in sterile females, and we therefore performed embryonic RNAi to examine gene function. In *Tc ct* RNAi larvae, all types of ESOs are affected (Figs. 5c and 6h–j). However, compared to the *Tc ASH* phenotype, most of the sensilla are present in *Tc ct* RNAi larvae but they show differentiation defects. We categorised these defects into ‘duplicated sensilla’, ‘reduced length of sensilla shaft’, ‘only socket of sensilla present’, ‘sensilla missing’, and ‘wildtype’ (Fig. 5c). The TSOs are most strongly affected in *Tc ct* RNAi. On the head, the ant_TSOs are affected in 94.12% of the cases, while 89.22% of the abdominal TSOs that show abnormal shape (Fig. 5c, turquoise). The predominant phenotype for all TSOs is a reduction in sensilla lengths (76.47% of ant_TSOs and 36.76% of abdominal TSOs; Fig. 6h, j, yellow arrows and circles).

The CSMs are the second most affected ESO subtype in *Tc ct* RNAi larvae: 58.24% show a phenotype in the head and 50.98% in the thorax (Fig. 6h, i; blue circles). There is a difference in the distribution of the sensilla phenotypes between head and thorax. In more than half of the affected CSM sensilla positions in the head, sensilla are absent (29.41%), while reduced length of sensilla shaft is the predominant phenotype in the thoracic CSMs (44.12%, Fig. 5c). Furthermore, 52.57–52.94% of the thoracic and abdominal CSGs are affected (Fig. 6i). The most prominent phenotype is again the reduced length of the sensilla shaft (36.40–47.06%). However, the abdominal CSGs show the highest percentage of the ‘only socket of sensilla present’ phenotype (15.44%) compared to all other sensilla types (Fig. 5c, brown bars). The BSMs are about equally affected across all body parts (35.29% in the head and thorax, 43.14% in the abdomen; Figs. 5c and 6i, j). Again, there are slight differences in the distribution of the sensilla phenotypes. In the head, about one third of the BSMs are missing in the affected positions, while differentiation defects are predominant in the affected BSMs of the remaining body parts (Fig. 5c). In addition, tracheal pits are absent in 96.15% of the *Tc ct* RNAi larvae, which corresponds with the prominent circular expression of the gene in the areas where the tracheal pits develop (Fig. 3e, f, i, j, open arrowheads; Additional file 1: Figure S3b, c; Fig. 6i, j; black asterisks indicating missing tracheal pits) and is in line with a previous *Tc ct* RNAi study [52]. Ct function in tracheal development seems to be conserved in insects [53, 54]. Taken together, *Tc ct* is required to various degrees for the development of all ESO subtypes. The position-specific *Tc ct* RNAi phenotype suggests that local cues influence the differentiation of the sensilla either, for example, by modulating *Tc ct* expression or by providing additional differentiation factors that regulate sensilla morphogenesis.

In contrast to the *Tc ct* RNAi phenotype, only a small subset of sensilla is affected in *Tc poxn* RNAi larvae. In t1 to t3, the mechanosensory sensillum plCSM is duplicated (50.88%), and in the abdominal segments, the mechanosensory alBSM (55.92%) and the olfactory pvTSO1 (81.80%) show the same duplication phenotype (beige bars in Fig. 5d; double arrowhead in Fig. 6f, g; yellow dotted circles in Fig. 6g).

We also attempted to analyse a gene that is regulated by Poxn in *D. melanogaster*, *target of pox neuro* (*tap*) [30]; however, *Tc tap* RNAi larvae were not analysable because of gross morphological defects such as the absence of the abdomen or deformation of head and thorax. These results are in line with those of the iBeetle screen [49]. We also attempted to functionally analyse two additional members of the Atonal family, *Tc amos* and *Tc cato*. However, similar to *Tc tap* RNAi, severe structural defects made it impossible to analyse the sensilla pattern in *Tc cato* RNAi larvae.

## Discussion

We show here that the expression and role of the proneural genes *Tc ASH* and *Tc ato* and homologues of the *D. melanogaster* subtype identity genes are not limited to specific categories of sensilla. This suggests that, in contrast to *D. melanogaster*, the concept that the identity of categories of sense organs can be assigned to the function of single or combinations of transcription factors cannot be applied in *T. castaneum*. Regardless of how we categorise the sense organs—by morphology, by function, or both—there is no transcription factor code that can be aligned with a single category. In the following, we discuss how the functions of the sensory genes have diverged in homologous classes of *D. melanogaster* and *T. castaneum* sensilla and how these findings might contribute to our understanding of the evolution of sense organ diversity in arthropods.

### Proneural gene expression and sense organ subtype identity are not directly linked in *T. castaneum*

A striking discovery of our study is that in *T. castaneum*, a single proneural transcription factor, *Tc ASH*, is required for the formation of all morphological and functional classes of ESOs analysed. This includes olfactory trichoid sensilla (TSOs), gustatory chaetoid sensilla (CSGs), and two types of mechanosensory sensilla, chaetoid (CSMs) and basiconic (BSMs). In contrast, in *D. melanogaster*, available molecular data on adult sense organs on the thorax and wing show that Ac and Sc determine (largely redundantly) the identity of external mechanosensory (trichoid and chaetoid) and gustatory sensilla (chaetoid), excluding olfactory sense organs [55–59].

Similarly, the role of the proneural gene Ato has diverged in *D. melanogaster* and *T. castaneum*. In *D. melanogaster*, Ato is essential for the development of a subset of adult olfactory sensilla on the head, the coeloconic sensilla on the antennae, and the basiconic sensilla on the maxillary palps [60] but it is not required in the olfactory trichoid sensilla which are specified by *amos* [34]. In contrast, in *T. castaneum*, *Tc ato* is expressed in trichoid (ant_TSO, abdominal TSOs), basiconic (alBSM), and chaetoid sensilla (alCSM, plCSM, pdCSG2). However, *Tc ato* is only required for the formation of one of these sense organs, the ant_TSOs (96.72% ant_TSOs absent in *Tc ato* RNAi), while a specific external morphological phenotype was not observed in the remaining ESOs. This includes the abdominal TSOs, which co-express *Tc ASH* and are morphologically different from the ant_TSOs (longer and thinner), suggesting they might represent a subcategory of trichoid olfactory sensilla in the *T. castaneum* larva. Future analysis will show if *Tc ato* plays a role in the differentiation of the internal TSO cell types (e.g. neuron, sheath cell), which we have not analysed here. Alternatively, it acts redundantly to *Tc ASH* as a proneural gene to increase the robustness of the system, similar as *ac* in *D. melanogaster sc* positive SOPs [58]. The same could also apply for the remaining *Tc ato/Tc ASH* positive sensilla (plCSM, pdCSG2) that do not show a phenotype in *Tc ato* RNAi. Vice versa, *Tc ASH* could have a redundant role or could be involved in the differentiation of ant_TSO, where it is co-expressed with *Tc ato* and does not show an RNAi phenotype. In this context, it is interesting to note that in the *D. melanogaster* larva, which does not exhibit antennae, Ato and Sc are required in a unique composite olfactory/gustatory dorsal organ on the head [51]. Sc seems to control the development of the non-olfactory sensory neurons, which are absent in the *sc* mutant larva.

The requirement of Ato in antennal sense organs seems to be conserved in Mandibulata (insects, crustaceans, myriapods), since Ato is expressed during the development of diverse prominent olfactory sense organs at the distal antennal tip of two other arthropod taxa, the aesthetascs of crustaceans on the 1st pair of antennae and the cone sensilla of millipedes [9, 40]. This suggests that Ato was recruited for the formation of antennal olfactory sense organs at the same time as antennae evolved in the last common ancestor of the Mandibulata and that these sense organs then diverged to adapt to different habitats.

### The regulation of subtype identity genes has diverged in *T. castaneum*

In *D. melanogaster*, the determination of sense organ subtype identity seems to be a decision between two (or three) alternative developmental pathways. Misexpression and loss-of-function studies show that Ac-Sc promote external mechanosensory versus internal mechanosensory (chordotonal organ) fate, while Ato supports formation of olfactory versus gustatory/mechanosensory sensilla externally, in addition to internal mechanosensory organ versus external mechanosensory organ fate [17, 23, 24, 32, 61, 62]. Subtype identity genes acting downstream of the proneural genes such as *ct* and *poxn* play a central role in determining the alternative fates [38, 63–65].

We show here for the first time that *D. melanogaster* subtype identity genes are involved in sense organ development outside the dipterans suggesting that these genes belonged to the sense organ toolkit at least in the last common ancestor of insects if not beyond. We tested five *D. melanogaster* homologues for potential roles in subtype identity: *ct*, *poxn*, the neurogenin-related gene *tap*, and two additional members of the Ato family, *amos* and *cato*. However, we did not find a specific role of the analysable genes (*Tc ct*, *Tc poxn*) in subtype identity in *T. castaneum* since we neither observed a transformation of one sensilla type into another, nor loss or differentiation defects in specific morphological or functional sensilla classes.

*Tc ct* is expressed in all types of analysed sensilla, and the predominant phenotypes in *Tc ct* RNAi larvae are differentiation defects, such as shorter sensilla and missing sensilla shafts (‘sockets only’, Fig. 5). In *D. melanogaster ct* mutants, however, external larval and adult mechanosensory organs are transformed into chordotonal organs [38, 63, 65]. Furthermore, the observation that *Tc ct* is expressed in all sensilla types, including those that (co-)express *Tc ato*, implies that the sensory gene network has diverged in insects since *D. melanogaster* Ato executes its subtype identity by suppressing *ct* expression [32]. A co-requirement of *Tc ato* and *Tc ct* in sense organ development is supported by the fact that in *Tc ct* RNAi larvae, the antennal olfactory sense organ ant_TSO is affected to a similar degree as in *Tc ato* RNAi larvae (*Tc ct* RNAi, 94%; *Tc ato* RNAi, 97%). However, the predominant phenotype in *Tc ct* RNAi larvae is a reduction in sensilla lengths, rather than the loss of the sensilla, indicating that *Tc ct* is involved in the differentiation of ant_TSO. Furthermore, we observed a *Tc ct* RNAi phenotype in the remaining TSOs that co-express *Tc ato*, *Tc ct*, and *Tc ASH*; the abdominal plTSO; and pvTSO1 and 2. Although not substantially affected in *Tc ato* RNAi larvae, these olfactory sensilla show a high percentage of differentiation defects in *Tc ct* RNAi larvae (89%) and are frequently absent in *Tc ASH* RNAi larvae (74%). To conclude, in *T. castaneum*, *ct* does not confer subtype identity; rather, the gene product seems to play a role in sensilla morphogenesis in all sense organs.

Similarly, the requirement of *Tc poxn* is not restricted to a specific class of sensilla; however, like in *D. melanogaster*, the gene is only needed in a small subset of larval sensilla [66]. In *Tc poxn* RNAi larvae, all functional types of the analysed sensilla are duplicated: gustatory (plCSM), mechanosensory (alBSM), and olfactory (pvTSO1). This is in contrast to *D. melanogaster*, where Poxn is required in the class of polyinnervated gustatory sensilla in larvae and adults [66, 67]. In larvae, for example, three types of polyinnervated sensilla (two basiconic sensilla (‘kölbchen’) per thoracic hemi-segment; one campaniform sensillum (‘papilla’) and one trichoid sensillum (‘hair’) per abdominal hemi-segment) are transformed into monoinnervated mechanosensory organs in *poxn* mutants [66, 68]. The observed duplication in *Tc poxn* RNAi larvae might be either due to a duplication of the SOPs or within the SOP lineage. The latter is supported by a detailed study of the sensilla lineages in *D. melanogaster poxn* mutant larvae showing that Poxn is required in the progeny of the SOPs and regulates the number and types of cells produced by each secondary precursor [66]. The three sensilla types affected in *Tc poxn* RNAi larvae (plCSM, alBSM, and pvTSO) express *Tc tap* suggesting that *Tc tap* might be activated by *Tc poxn* and expressed in the same sensilla as it is the case in *D. melanogaster* larvae [30]. Functional studies of *Tc tap* could have contributed to our understanding of the regulatory mechanisms in *poxn* positive sense organs, but unfortunately, the *Tc tap* RNAi larvae were not analysable. Taken together, we found no obvious role of *Tc poxn* in subtype identity; rather, the duplication phenotype suggests a role in sensory organ cell lineage regulation.

We attempted to analyse the function of the Atonal family genes *Tc amos* and *Tc cato*, but severe overall morphological defects prevented us from studying the sensilla pattern. Based on our gene expression analysis, however, *Tc cato* does not show sense organ subtype-specific expression: it is expressed in all morphological and functional types of sensilla. Expression starts after formation of the SOPs, slightly earlier than that of *Tc ct*. Interestingly, in *D. melanogaster*, *cato* expression is only temporarily restricted to specific sense organ types. The gene is initially only expressed in the Ato- and Amos-dependent sense organs, but later expression appears also in the larval external mechanosensory sensilla, after *ac-sc* are switched off and after the start of *ct* expression [34].

### Evolutionary scenario

**Fig. 7:**
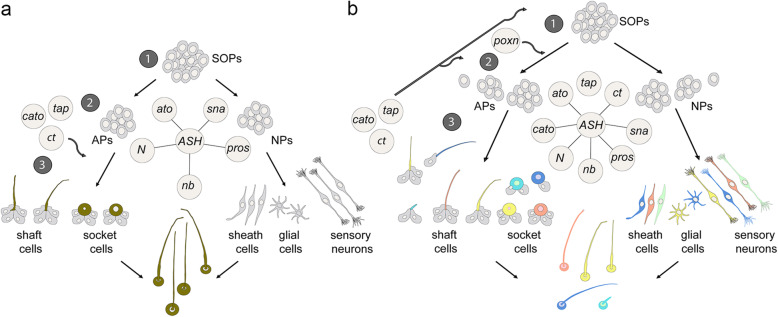
Evolutionary model of sense organ diversification in arthropods. The different colours represent additional diversity of morphology and function achieved by changes in the temporal expression and regulation of sensory genes. The numbers refer to the subsequent processes of sense organ development. See text for details. APs, accessory cell precursors; NPs, neural precursors

In the following, we discuss a model that explains how the mechanisms underlying sense organ diversification in arthropods might have evolved. Sensory genes are active at different time points and thus can be assigned different roles in the process of subtype identity development according to their expression and mutant phenotypes: (1) formation and subtype specification of SOPs, (2) specification of accessory (e.g. bristles, sockets) and neural cell types, and (3) differentiation of sense organ cells (Fig. 7).

In all arthropod species analysed, *ASH* is at the top of the sensory gene cascade and shows a widespread early expression in different morphological and functional types of sensilla prior to or during formation of the SOPs [9, 40, 69]. We therefore suggest that in the last common ancestor of arthropods, *ASH* was the predominant proneural gene for sense organ development and that ASH endowed epidermal cells with the potential to develop into external sense organs *without* simultaneously specifying subtype identity (Fig. 7a). The presence of an *Ato-like* gene in prebilaterian sense organs [70] suggests that *ato* belonged to the sensory toolkit of the arthropod ancestor together with ‘general’ cell fate specification and differentiation genes that are expressed in all sense organs in all arthropod species that have been analysed so far (*sna*, *pros*, *N*, *nb*; e.g. [9, 39, 40, 69]). Since members of both protostomian phyla (Ecdysozoa and Lophotrochozoa) possess functionally diverse sense organs (e.g. [10, 71]), we can assume that the last common ancestor of arthropods started out with some diversity of sensory neurons and accessory cells (Fig. 7a). Based on studies in arthropod groups other than insects, the crustaceans, myriapods, and chelicerates, we can furthermore assume that the ancestral sense organs in the last common ancestor of the euarthropods developed from groups of SOPs, rather than single SOP lineages as seen in dipterans [9, 40, 69].

Initially, genes that were added to sensory development might have appeared late in development and were required for regulating neuronal differentiation such as axonal projections and the structure of dendrites (Fig. 7a). The increase of the sensory gene toolkit might have been supported by lineage-specific duplications of genes already part of the network, such as ASH and Atonal family members [27, 34, 72]. An example is the lineage-specific expansion of atonal genes in mosquitoes who harbour three *atonal* genes in their genome in contrast to most other insects [73]. In the next step, changes in the temporal expression of the add-on genes changed their role and importance in sense organ development (Fig. 7b). Other genes might have been directly recruited to the earlier process of accessory (socket, shaft, sheath cells) and neuronal cell type specification (neurons, glia) to regulate the developmental potential and divisions of the secondary precursors (e.g. *poxn*). The addition of sensory genes to the existing set resulted in variations in cell type numbers and morphology that were used as an evolutionary tool for sense organ diversification (Fig. 7b).

This evolutionary scenario is supported by variations in the roles, regulation, and temporal expression of sensory genes observed in different sense organs and species. *tap*, for example, which is expressed late during the development of a subset of sense organs in *T. castaneum* and in gustatory sense organs in *D. melanogaster*, is expressed at the time of SOP formation in the *D. melanogaster* adult antennal olfactory sensilla [31]. Similarly, *D. melanogaster cato* shows variations in its temporal expression, ranging from SOP formation to terminal differentiation in different chordotonal organs and ESOs [74, 75]. Furthermore, Ct, which is expressed in ESO SOPs, has a late role in dendritic arborisation in a subtype of multidendritic (MD) neurons [76]. These examples indicate that the sensory gene network is modified locally by spatial and temporal cues, which has indeed been demonstrated in *D. melanogaster* (e.g. [37, 77, 78]). Similar context-dependent mechanisms might operate in *T. castaneum*. For example, the *Tc ASH-Tc ato* positive sense organs, which occupy the same position directly opposite the tracheal pits in all trunk segments, develop into the gustatory plCSMs in the thoracic segments but into the olfactory plTSOs in the abdominal segments (Fig. 2d). The potential for spatial and temporal modulation of sense organ development opens up an additional evolutionary route for generating sensory diversity and provides an explanation for the lack of sense organ subtype specificity of the genes analysed here: proneural genes might be used interchangeably for different subtypes because their target gene specificity is modulated by positional cues. Thus, a conserved sensory gene network can be used flexibly in different contexts to generate diversity.

Further evolution of sense organ specification leading to the *D. melanogaster* model, where sense organs develop along alternative pathways, might have been supported by changes in the regulation of the sensory genes. This assumption is supported by variations in the co-expression of genes in different arthropod groups and sense organs. In representatives of crustacean and myriapods [9, 40] and in *T. castaneum* (this study), co-expression of *Tc ASH* and *Tc ato* has been observed in many sense organs. In addition, *Tc ct* is co-expressed both with *ato* and *ASH*. In contrast in *D. melanogaster*, both Amos and Ato execute their subtype identity role by suppressing *ac-sc* and *ct*, respectively [32, 37].

### How did the urbilaterian specify its sense organs?

The proposed evolutionary model is based on comparative studies in arthropods and suggests that in their last common ancestor *ASH* was the predominant proneural gene and that promotion of neurogenesis (i.e. generation of SOPs) was not directly coupled with subtype identity. Does this ancestral state apply to arthropods only or can similarities be traced further back to the last common ancestor of bilaterians, the urbilaterian?

*ASH* and *Atonal-like* genes are part of the developmental toolkit of sensory cells in prebilaterians (e.g. sponges: [79]; e.g. cnidarians: [70, 80, 81]). In cnidarians, an *Atonal/Neurogenin* homologue is expressed more widely and earlier than *ASH* indicating that ASH is involved in differentiation of sensory cells, rather than determination of SOPs [70, 81]. Furthermore, *Atonal/Neurogenin* is not the first neural gene to be expressed in the progenitors: it is expressed after *SoxB* [70], suggesting that the gene product is required for the execution of the sensory programme, rather than the initial neural cell fate determination. All three gene families are also expressed in sense organs of representatives of Protostomia and Deuterostomia (e.g. [70]), additionally confirming that they must have belonged to the sensory gene network of the urbilaterian.

Furthermore, the gene families were likely able to support distinct proneural activities in the urbilaterian. This is supported by previous studies that replace the *D. melanogaster ato* coding sequence (CDS) with the CDSs of *Ato/Neurogenin* members from across the metazoans (mouse, lancelet, annelid, sponge), including the CDSs of the *D. melanogaster* Achaete-Scute Complex as outgroup [82]. While the *Ato* members and the *Ato/Neurogenin* sponge gene could rescue the *D. melanogaster ato* mutant sense organ phenotype, the neurogenins could not replace *D. melanogaster ato* function and neither could the members of the Achaete-Scute Complex.

A comparison of the role and expression of vertebrate proneural genes together with the data from prebilaterians [70] agrees with our model of generating diversity by shifts in the temporal expression of sensory genes. For example in vertebrates, cranial ganglion sensory neurons are specified by the *ato-related* gene NeuroG [83], while in the olfactory epithelium of the mouse, *Ascl1* (formerly *Mash1)* is expressed first, followed by *Math4C/neurogenin* [84]*.* In both cases, NeuroD, another *atonal-like* gene, is expressed during differentiation of the sensory neurons. In the otic placode, *neurogenin 1* is expressed at the top of the hierarchy in the neural progenitor and then in the sensory neuron together with *NeuroD*, followed by *Math1* expression in the mechanosensory hair cell, a distinct ear cell type [85, 86]. These data are again in line with our findings in the *T. castaneum* showing that proneural families can support diverse sensory functions and morphologies.

Furthermore, it can be assumed that the function of proneural genes of simultaneously promoting neural fate and subtype identity was uncoupled in the urbilaterian. This assumption is in line with the data presented here and supported by the mechanisms of sensory neuron specification in the sensory placodes of vertebrates. For example, in the olfactory placode, proneural genes are not expressed in the olfactory stem cells, which give rise to diverse neurons; rather, *Ascl1* is expressed in the so-called transit amplifying neuronal progenitors that have progressed from the multipotent precursor state and the gene is required for the expansion of the olfactory receptor neurons [87]. Subtype identity of the different olfactory neuron populations (olfactory receptor neurons, vomeronasal receptor neurons, and gonadotropin releasing hormone neurons) is achieved by inductive signalling from the underlying mesenchyme (e.g. [88, 89]).

## Conclusions

Taken together, our results support an evolutionary scenario whereby sensory genes are recruited to the development of individual or subsets of sense organs, which do not necessarily fall into specific morphological or functional classes. Changes in the temporal expression can move the genes up in the hierarchy so that they can control all aspects of a specific sense organ subtype, as is the case for *ct* and *poxn* in *D. melanogaster*, for example. The *T. castaneum* data presented here also fit this evolutionary scenario. *Tc ct* has been recruited to a late step of development in all sense organs, regulating the differentiation of shaft cells, while *Tc* Poxn is only required in a subset of sensilla possibly controlling cell type numbers. *Tc cato* is expressed earlier than *Tc cut* and might therefore be involved in sensory organ cell type specification, similar to *Tc poxn* but in all sense organs. None of these genes are expressed during formation of the SOPs and therefore do not have control over the whole specification process, i.e. sense organ subtype identity.

To conclude, the evolutionary scenario presented here suggests that sense organ subtype identity has evolved by recruitment of a flexible sensory gene network to the different sense organ specification processes. A dominant role of these genes in subtype identity has evolved as a secondary effect of the function of these genes in individual or subsets of sense organs. Positional cues must have had a major influence on the evolution of the subtype identity, and corresponding spatial and temporal enhancers have indeed been identified in proneural genes, e.g. [90, 91]. Future comparative analysis will show how patterning mechanisms have shaped the evolution of sense organ diversity.

## Methods

### Animal husbandry

*T. castaneum* beetles were reared as previously described [47]. The San Bernadino (SB) wildtype strain was used for all experiments.

### Sequence analysis and cloning of *T. castaneum* genes

*Tc ASH*, *Tc ato*, *Tc poxn*, *Tc cato*, *Tc tap*, and *Tc amos* gene sequences were obtained from the iBeetle-Base [92, 93]. A phylogenetic tree using ClustalW alignment of selected bHLH containing protein sequences (*ato*, *cato*, *amos*) was generated (see Additional file 1: Figure S6 [94], Table S7 for sequence accession numbers) to demonstrate correctness of annotation. Sequences were amplified from cDNA (synthesised from extracted RNA from different developmental stages with SuperScript III First-Strand Synthesis System (Invitrogen) and cloned into pGEM®-T Easy Vector (Promega) using standard cloning procedures (Additional file 1: Table S8 for primer information)). Sanger sequencing (performed by Eurofins) was used to confirm sequences and orientation of fragments in the plasmid.

### mRNA probe synthesis and in situ hybridisation

Antisense mRNA probes for genes in this study were synthesised from their cloned sequences (as described above). The in vitro transcriptions using the T7 RNA polymerase and the DIG RNA labelling Mix (both Roche) were performed following the supplier’s protocol. Colorimetric whole mount in situ hybridisation (NBT/BCIP) was performed as previously described using anti-DIG antibody conjugated with Alkaline-Phosphatase (Roche) [95].

### RNA interference

Parental RNAi (pRNAi) (*Tc ASH*, *Tc ato*, *Tc poxn*, *Tc cato*, *Tc tap*, *Tc amos*) and embryonic RNAi (eRNAi) (*Tc ct*) were performed to infer gene functions. Double-stranded RNA (dsRNA) for all genes was ordered from Eupheria Biotech (Dresden, Germany; based on sequences from the iBeetle-Base [92, 93]). For each gene, two non-overlapping fragments (NOF1 and NOF2) were injected (where NOF1 is the same as the iB-RNA fragments used in the iBeetle screen [49]; see Additional file 1: Table S9 [49] and Figure S7 for list of iB-RNA numbers and location). For pRNAi, female pupae were injected with 1 μl/μg dsRNA as previously described ([96–98] and ‘The Beetle Book’, http://wwwuser.gwdg.de/~gbucher1/tribolium-castaneum-beetle-book1.pdf). Embryos were injected with 3 μl/μg dsRNA as previously described [99]. The protocol was adjusted slightly. Embryo preparation, mounting, and injection were performed as described [99]. However, after injection, the coverslips with the embryos were placed upside down onto the oxygen permeable membrane with 3 stacked coverslips used as bridges, and the intervening space was filled with halocarbon oil. This petri dish set-up was inverted and placed on a layer of 1% agarose gel to maintain humidity. The embryos were kept in this condition at 32 °C until the embryos hatched. For pRNAi and eRNAi experiments, negative controls were either injected with H_2_O or injection buffer (1.4 mM NaCl, 0.07 mM Na2HPO4, 0.03 mM KH2PO4, 4 mM KCl, pH 6.8).

### Quantification of RNAi phenotypes and statistical analyses

First instar larvae (L1) of pRNAi and eRNAi experiments were used for cuticle analysis and prepared as described before [100]. L1 cuticle preparations were analysed for wildtype larvae (wt), ‘phenotype’, ‘non-specific’ (i.e. broken cuticles, larvae still in egg membrane and hence sensilla not analysable, etc.), and ‘empty eggs’ (results are summarised in Additional file 1: Table S6). The larvae showing a phenotype were screened for the sensilla in prominent positions described in Fig. 1. These sensilla were counted in each larva on one side (preferably right side) and categorised, where applicable, as ‘wildtype’, ‘missing sensilla’, ‘duplicated sensilla’, ‘reduced length of sensillum shaft’, and ‘only socket of sensillum present’. The recorded data for each gene are summarised for dsRNA NOF1 and NOF2, and further categorised into sensilla subtypes (TSOs, BSMs, CSMs, and CSGs) and body parts (head, thorax, abdomen). An overview of the recorded data can be found in Additional file 1: Tables S1-5. Microsoft Excel was used to document and process data for statistical analysis.

### SEM sample preparation

*T. castaneum* 1st instar larvae (24 h egg lays were incubated at 32 °C for 3 days) were collected and washed with PBS. The washed larvae were fixed in 1:1 heptane and 3% glutaraldehyde at room temperature for 1 h. The fixative was removed, and the larvae were dehydrated with a series of acetone (70%, 80%, 2× 90%, 2× 100%). HMDS was used to dry the larvae (larvae were incubated in HMDS, and then, the solution was removed, followed by air drying larvae in a block dish overnight under the fume hood). The dried larvae were mounted on aluminium stubs with sticky tape and sputter coated with gold (Agar auto sputter coater model 108A). A FEI Quanta 3DFEG or a FEI Inspect F electron microscope was used for imaging L1 larvae visualising sensilla morphology.

### Microscopy and image processing

Following in situ hybridisation (colorimetric), the yolk of *T. castaneum* embryos was removed. The embryos were then mounted flat onto microscope slides using glycerol. Colorimetric stained embryos (NBT/BCIP staining reaction) as well as cleared cuticles of *T. castaneum* larvae were imaged, or screened using an inverse Leica microscope (DM IL, Wetzlar, Germany) and corresponding LAS software (version 2.8.1). Image acquisition of L1 cuticles was performed with a Leica SP5 confocal microscope (Wetzlar, Germany) and corresponding LAS X software. Z-stacks of L1 confocal images were processed using the Z-projection tool of Fiji [101]. Graphic design programs (Adobe photoshop CS2, Adobe illustrator, and Inkscape version 0.92) were used for image processing, assembly, and preparations of schematic illustrations.

## Supplementary Information

**Additional file 1: **‘Functional analysis of sense organ specification in the *Tribolium castaneum* larva reveals divergent mechanisms in insects’: Supplementary figures and tables. The file contains **Figures S1-S7** and **Tables S1-S9** in pdf format.

## Data Availability

All data generated or analysed during this study are included in this published article and its supplementary information files.
