# Supplementary material for: Functional analysis of sense organ specification in the Tribolium castaneum larva reveals divergent mechanisms in insects
Source: BMC Biol. 2021 Feb 5;19:22. doi: 10.1186/s12915-021-00948-y (PMC7866635; doi:10.1186/s12915-021-00948-y)
Supplement: Supplementary file 1 — Additional file 1: ‘Functional analysis of sense organ specification in the Tribolium castaneum larva reveals divergent mechanisms in insects’: Supplementary figures and tables. The file contains Figures S1-S7 and Tables S1-S9 in pdf format. [file 12915_2021_948_MOESM1_ESM.pdf]

Additional file 1

Functional analysis of sense organ specification in the *Tribolium castaneum* larva reveals divergent mechanisms in insects

Marleen Klann[1,2*], Magdalena Ines Schacht[1*], Matthew Alan Benton[3], Angelika Stollewerk[1]

1 School of Biological and Chemical Sciences, Queen Mary University of London, Mile End Road, London E1 4NS, United Kingdom

2 Marine Eco-Evo-Devo Unit, Okinawa Institute for Science and Technology (OIST), 1919-1 Tancha, Onna-son, Okinawa, 904-0495 Japan

3 Department of Zoology, University of Cambridge, Downing St, Cambridge CB2 3EJ, United Kingdom

*contributed equally

Corresponding author: Angelika Stollewerk (a.stollewerk@qmul.ac.uk)

Additional file 1: Supplementary Figures S1-S7 and Supplementary Tables S1-S9.

Supplementary Figures

Figure S1 *Tribolium castaneum* staging system

Figure S2 Comparison of *Tc ASH* and *Tc ato* expression patterns

Figure S3 Expression patterns of *Tc ct, Tc cato* and *Tc tap*

Figure S4 Expression patterns of *Tc ase*, *Tc pros*, *Tc sna*

Figure S5 *Tc ASH* RNAi phenotypes

Figure S6 Phylogenetic analysis of Atonal family proteins

Figure S7 Maps of mRNA architecture of analysed genes

Supplementary Tables

Table S1 Variations in sensilla numbers in the negative control

Table S2 *Tc ASH* RNAi quantification of phenotypes

Table S3 *Tc ato* RNAi quantification of phenotypes

Table S4 *Tc ct* RNAi quantification of phenotypes

Table S5 *Tc poxn* RNAi quantification of phenotypes

Table S6 Summary of RNAi injection results for both dsRNA fragments

Table S7 List of analysed Atonal family proteins

Table S8 Primer sequences

Table S9 Double-stranded RNA information

Supplementary Figures

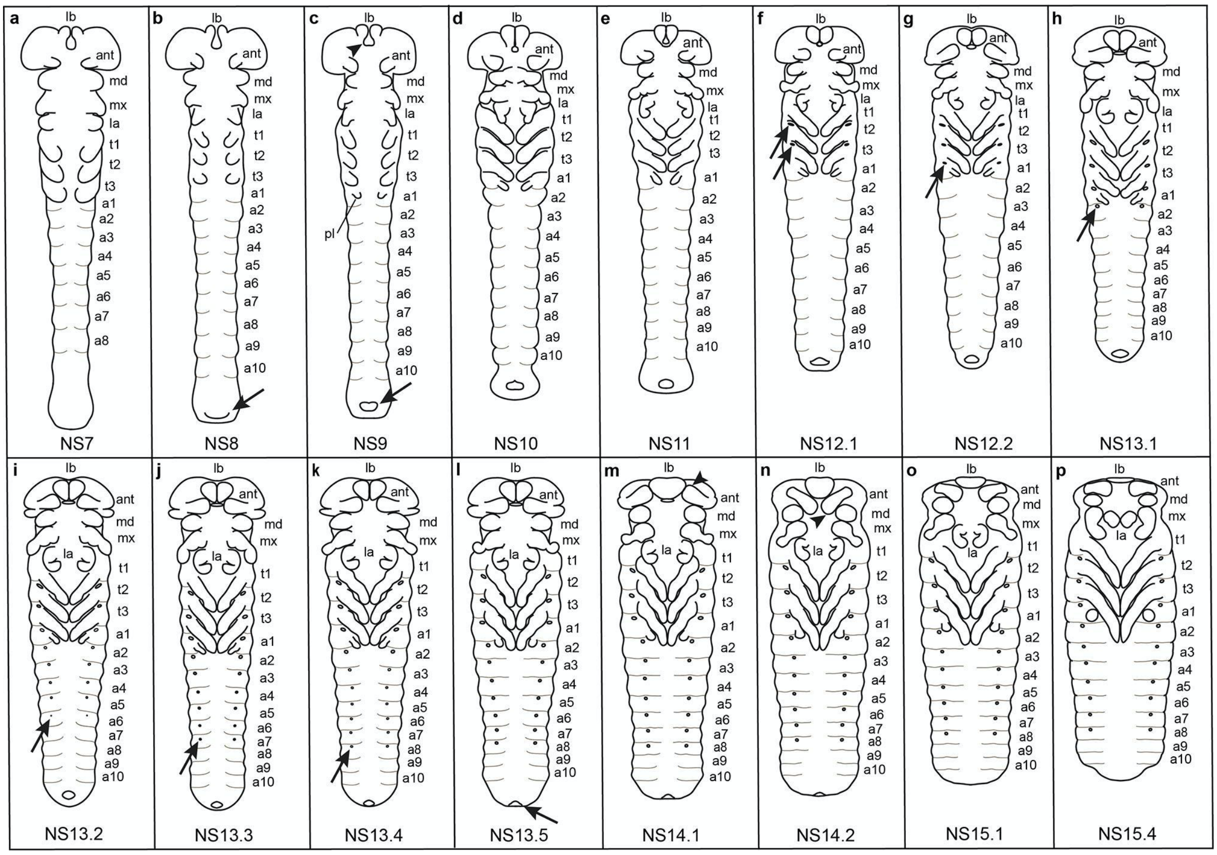

Figure S1. *Tribolium castaneum* staging system. Schematic drawings based on light microscopic images of flat preparations. In *T. castaneum,* the phases of embryonic development are usually subdivided into hours of development after egg laying (AEL) at 32 °C [44-46]; development takes approximately 70h at 32°C [47]. The presented morphological staging system is based on Biffar and Stollewerk [43] (NS1 to NS15 corresponding to 9h to 52h AEL at 32°C), including additional subdivisions of later stages (NS12-15). Stages relevant to sense organ development: NS7-15. (a) NS7 (18h): limb buds, t1-3 and a1-8 visible; triangular shape of maxillae, labrum: paired lobe; (b) NS8 (20h): a1-10 and proctodeum (arrow) visible; elongation of thoracic legs. (c) NS9 (22h): greatest length of embryo; labrum and stomodeum: triangular shaped (arrowhead); pleuropodia visible; proctodeum: round shape (arrow). (d) NS10 (24h): further elongation of legs; indentations on mandible, maxilla, labium indicate podomere formation; two lobes of the labrum aligned along their proximo-distal length. (e) NS11 (26h): further elongation of appendages; labium: hook shaped. (f) NS12.1 (28h): condensation of the segments; tracheal pits on t2-3 (arrows). (g) NS12.2 (30h): antennal tips point upwards; tracheal pits on a1 (arrow). (h) NS13.1 (32h): further elongation of legs; tracheal pits on a2 (arrow). (i) NS13.2 (34h): tracheal pits up to a6 (arrow). (j) NS13.3 (36h): tracheal pits on a1-7 (arrow). (k) NS13.4 (38h): tracheal pits on a1-8. (l) NS13.5 (40h): proctodeum (arrow) at more dorsal position. (m) NS14.1 (42h): antennae point upwards (arrowhead). (n) NS14.2 (44h): antennae point downwards (arrowhead). (o) NS15.1 (46h): antennae point

towards the ventral midline; labrum length reduced; embryo shortens further. (p) NS15.4: embryo shortens further; maxillae: hook shaped; labia located between maxillae. a1-8, abdominal segment 1-8; ant, antennal segment; la, labial segment; lb, labrum; md, mandibular segment; mx, maxillary segment; t1-3, thoracic segment 1-3.

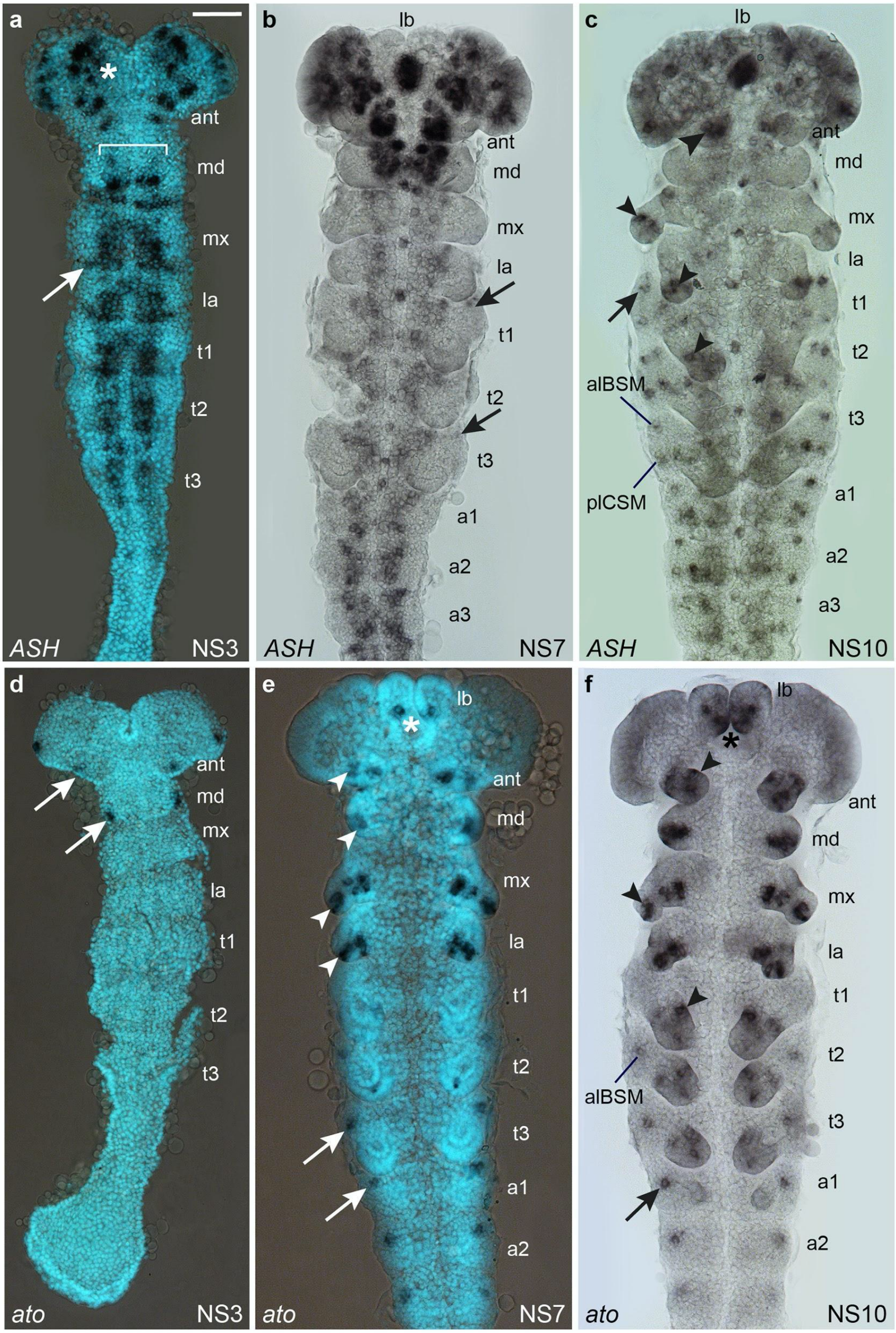

Figure S2. Comparison of *Tc ASH* and *Tc atonal* expression patterns in the peripheral nervous system. Light (b, c, f) and fluorescence (a, d, e) micrographs of flat preparations

stained with DIG labelled RNA probes and SYBR Green (light blue; a, d, e). Anterior is towards the top. (a) NS3: *Tc ASH* is expressed in the central nervous system (CNS; asterisk) and ventral neuroectoderm (VNE, bracket). In t1-3, expression extends laterally forming a small stripe (arrow). The stripes disappear before clusters of cells emerge where sense organs form. (b) NS7: *Tc ASH* expression is visible in a few cells in the lateral body wall (arrows). The gene is also expressed in the developing brain and VNE. (c) NS10: *Tc ASH* expression has increased in the peripheral nervous system (PNS) and is visible in single and groups of cells in the lateral body wall (arrow). Based on the expression with regard to landmarks, the expression domains correspond to the developing alBSM (anterior-lateral basiconic seta, mechanosensory) and plCSM (posterior-lateral chaetoid seta, mechanosensory). *Tc ASH* domains are also visible in the antennal (large arrowhead), maxillary, labial and thoracic appendages (small arrowheads). (d) *Tc ato* is first expressed at stage NS3 in the antennal and mandibular segments (arrows). The bilateral domains in the head lobe most likely correspond to the developing optic lobes. (e) NS7: *Tc ato* expression is visible in all head appendages (arrowheads) and the labrum (asterisk), and starts in the lateral body wall (arrows). (f) NS10: the same expression domains are visible in the elongated head appendages (arrowheads), the labrum (asterisk) and the lateral body wall (arrows). The expression in the lateral body wall most likely corresponds to alBSM. *Tc ato* domains are visible in the thoracic appendages. Abbreviations see Fig. S1. Scale bar: 50 µm.

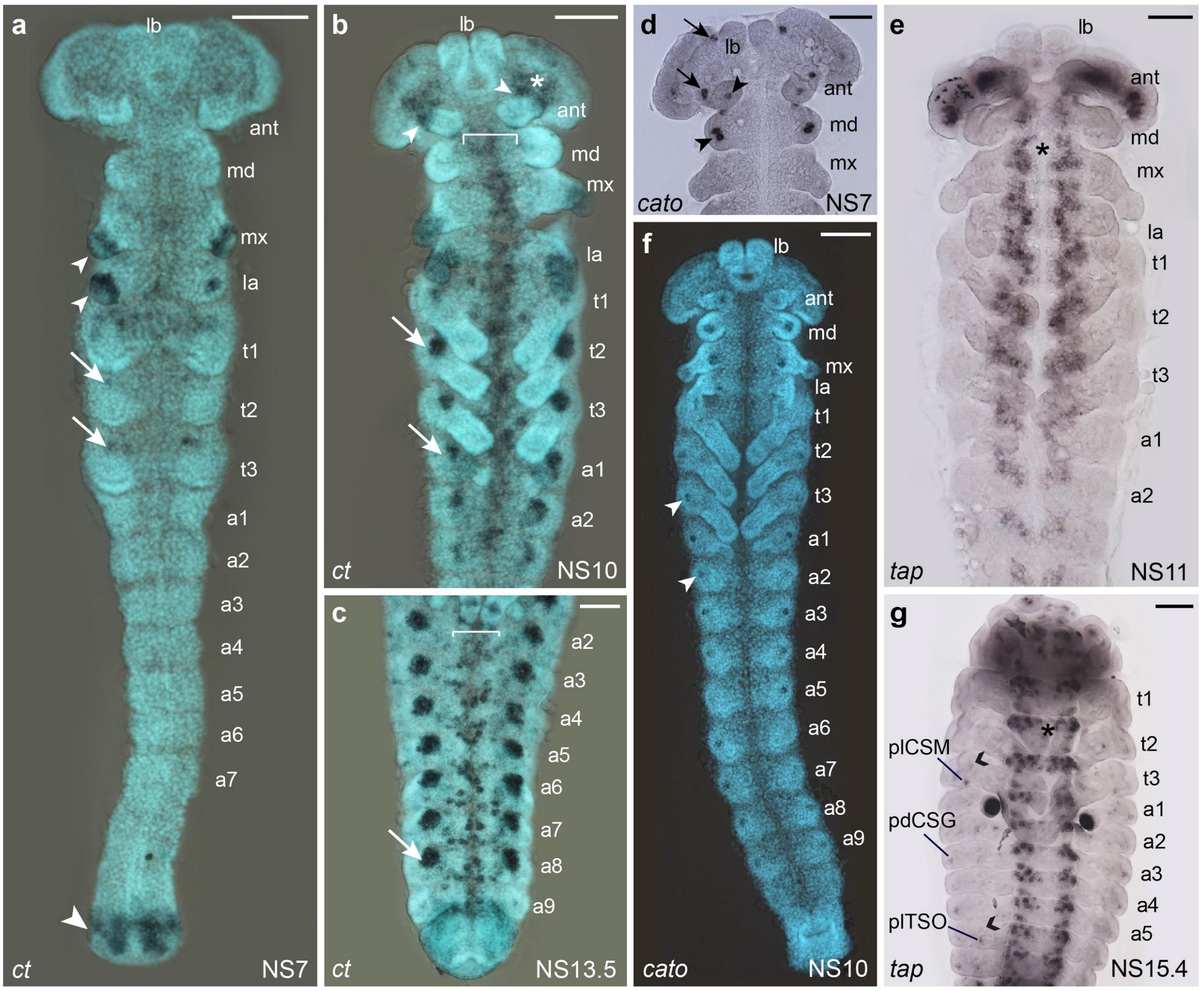

Figure S3. Expression patterns of the sense organ subtype specific genes *Tc ct, Tc cato* and *Tc tap.* Fluorescence micrographs of flat preparations stained with DIG labelled RNA probes (dark blue) and SYBR Green (light blue; a-c, f) and light micrographs stained with DIG labelled RNA probes (d, e, g). Anterior is towards the top. (a) *Tc ct* expression is first visible at NS7 at the tips of the maxillary and labial appendages (small arrowheads) and around the proctodeum (large arrowhead). Small expression domains are visible in the areas where the tracheal pits form (arrows). (b) NS10: *Tc ct* is expressed at the base of the developing antennae (arrowheads) and the whole tips of the maxillary and labial appendages. In addition, *Tc ct* expression is visible in the brain (asterisks) and the developing ventral nerve cord (bracket). *Tc ct* is also strongly expressed in the developing tracheal pits of t2-3 and a1-3 (arrows). (c) NS13.5: *Tc ct* expression is visible in the VNE (bracket). The expression around the tracheal pits has extended to a8 (arrow). (d) NS7: *Tc cato* is expressed in the antennal and mandibular appendages (arrowheads). Additional expression is visible both at the base of the labrum and the antennae (arrows). (e) *Tc tap* expression starts in the CNS (asterisk) at NS11. (f) *Tc cato* is first expressed in the lateral body wall in single clusters (arrowheads) at NS10. (g) NS15.4: *Tc tap* expression has decreased and is only detectable in a few cells corresponding to the positions of plCSM, one of the two abdominal pdCSGs and plTSO. The

open arrowheads point to the tracheal pits. Abbreviations see Fig. S1. Scale bars in a, b, g, f 100 µm; c-e, 50 µm.

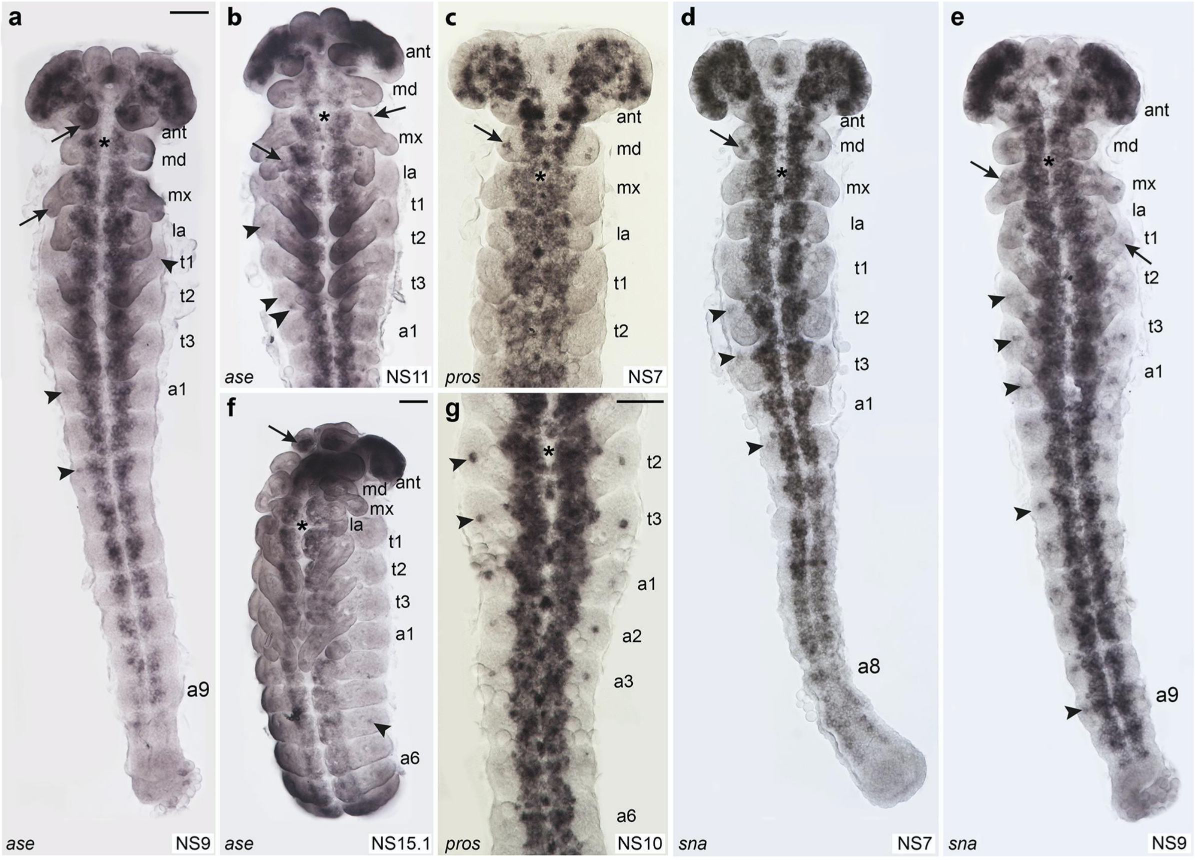

Figure S4. Expression patterns of the panneural genes *Tc asense*, *Tc prospero* and *Tc snail*. Light micrographs of flat preparations stained with DIG labelled RNA probes of *Tc ase*, *Tc pros* and *Tc sna*, respectively. Anterior is towards the top. The asterisks indicate the expression of the genes in the CNS. (a) NS9. *Tc ase* is expressed in the head appendages (arrows) and in a few cells in the lateral body wall (arrowheads). (b) The latter expression persists into NS11 (small arrowheads). Additional cells express *Tc ase* in the appendages (arrows) and the lateral body wall (large arrowhead). (c) NS7: *Tc pros* is expressed in the mandibular appendages in addition to the CNS. (d) *Tc sna* expression is visible in the same domains as *Tc pros* in the developing mandibles (arrow) at NS7. In addition, *Tc sna* positive cell clusters appear in the lateral body wall of t2, t3 and a1-2 (arrowheads). (e) NS9: this expression has extended up to A9 (arrowheads). In addition, *Tc sna* is expressed in clusters in all appendages (arrows). (f) NS15.1: *Tc ase* expression has decreased in the PNS except for a few cells in the lateral body wall (arrow) and a domain at the tip of the antennae (arrow). (g) NS10: *Tc pros* is expressed in clusters of cells in the lateral body wall of t2, t3 and a1-a3. Abbreviations see Fig. S1. Scale bar in a, 50 µm in a-e, scale bar in f, 100 µm, scale bar in g, 50 µm.

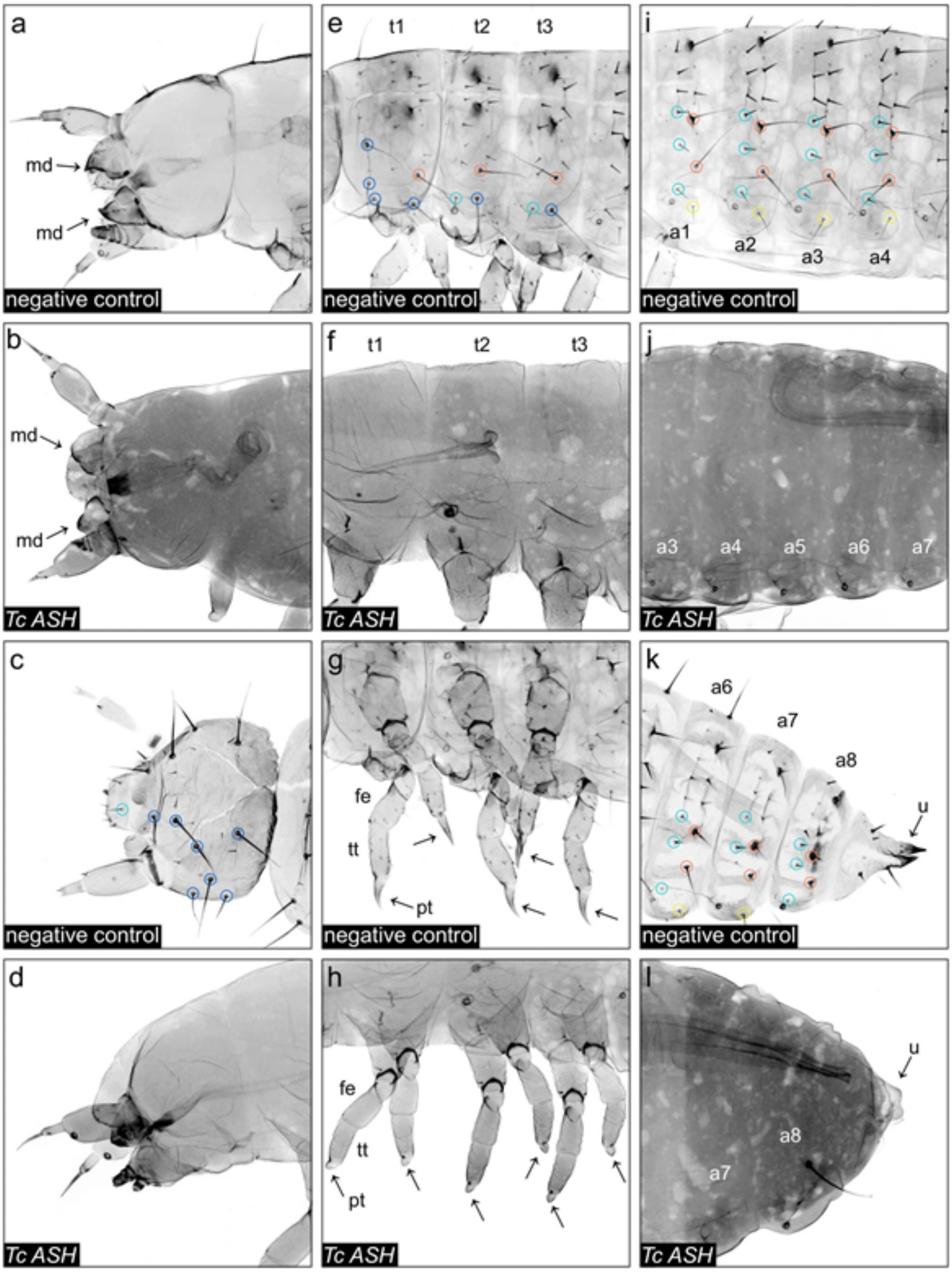

Figure S5. *Tc ASH* RNAi phenotypes. Confocal images of larval cuticles 1st larval stage). Anterior is to the right. (a, c, e, g, i, k) Negative control cuticles. Please note that the sensilla of the head capsule and thorax are out of focus. (b, d, f, h, j, l) *Tc ASH* RNAi cuticles. (b, f, j, d) The strongest cuticle phenotype resulting from *Tc ASH* RNAi are 'naked' larvae, i.e. larvae missing all external sensilla (compare to c, e, i). (b, h, l) *Tc ASH* RNAi also leads to rounded pretarsi (pt) (47.89% (n=263) have at least one pretarsal segment rounded), urogomphi (u)

(100% (n=263) have rounded u), and mandibles (md) (83% (n=263) have rounded md) (arrows; compare to (a, g, k)). Scale bar, 50 μm.

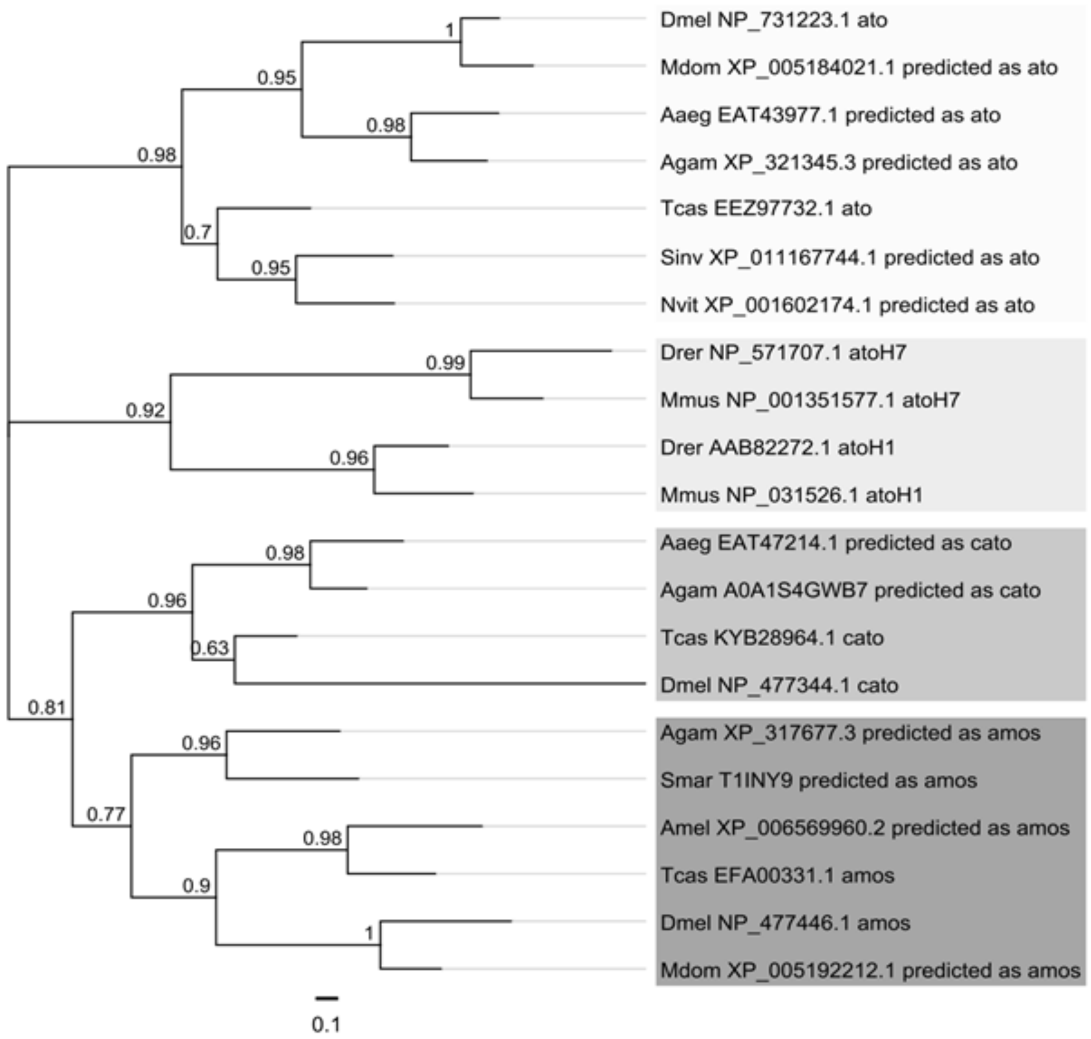

Figure S6. Phylogenetic analysis of Atonal family proteins. Maximum-likelihood analysis of full-length predicted protein sequences (*atonal* (*ato*), *cousin of atonal* (*cato*), and *absent MD neurons and olfactory sensilla* (*amos*)) in different insect species and two vertebrate species. Four different clusters can be identified, one insect specific *ato* cluster (containing *Tc ato*), one vertebrate specific *ato* cluster, and one each for insect specific *cato* and *amos* proteins. Branch labels are FastTree support values [94]. *Aaeg, Aedes aegypti; Agam, Anopheles gambiae; Amel, Apis mellifera; Dmel, Drosophila melanogaster; Drer, Danio rerio; Mdom, Musca domestica; Mmus, Mus musculus; Nvit, Nasonia vitripennis; Sinv, Solenopsis invicta; Smar, Strigamia maritima; Tcas, Tribolium castaneum.* Gene names (predicted) and NCBI accession numbers are indicated. Note that *Smar amos* ID (T1INY9) is from UniProt. (See Table S7 for list of sequences).

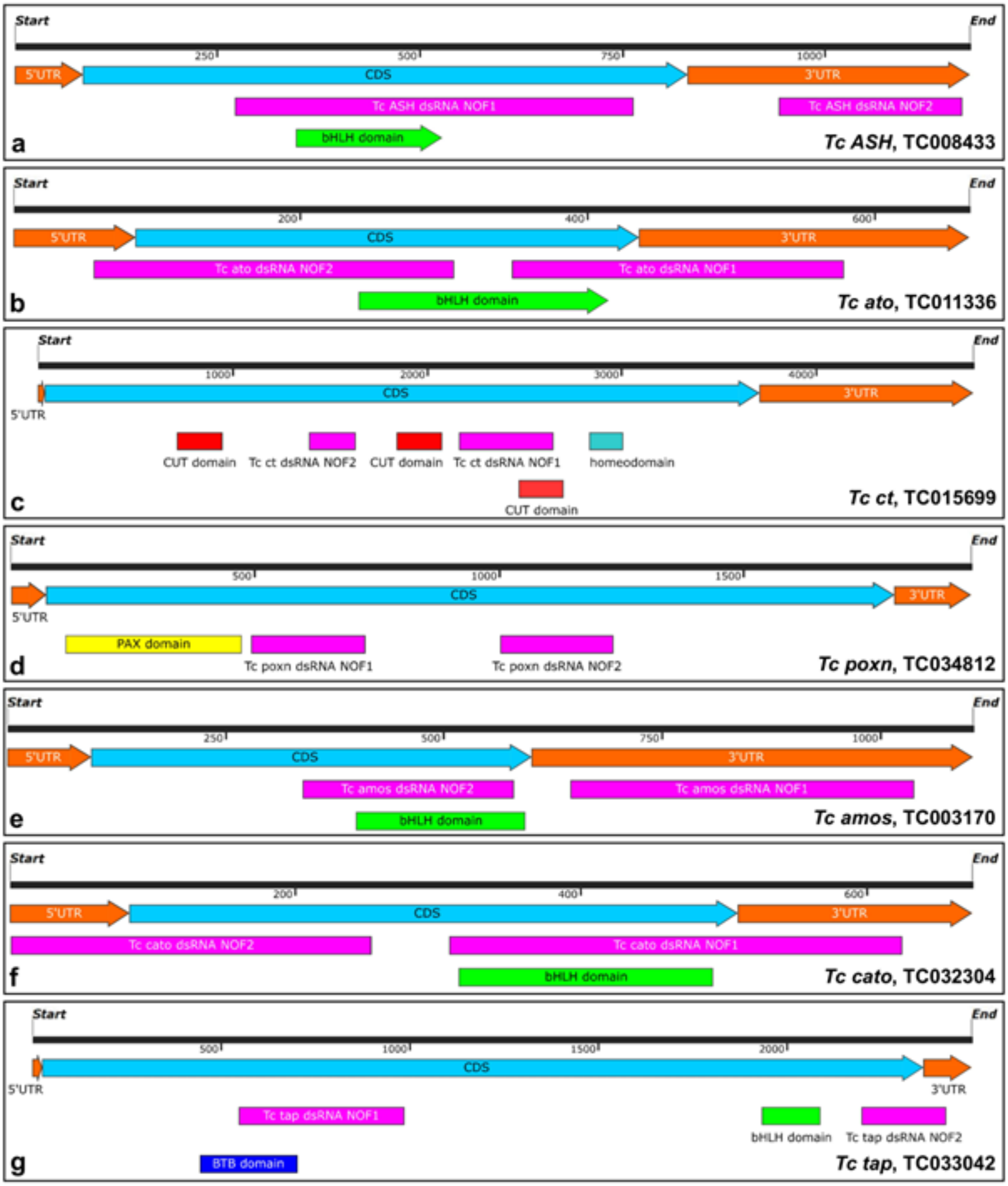

Figure S7. Maps of mRNA architecture of analysed genes. (a-g) Each panel shows 5’ and 3’UTR (orange) and coding sequences (blue), as well as location of dsRNA NOFs (pink), and the location of the conserved DNA binding domains (in green (bHLH), red (CUT domain), turquoise (homeodomain) and yellow (Pax domain). Gene names and corresponding TC sequence numbers are given in the bottom right corner of each panel. Maps were generated with the SnapGene software (from Insightful Science)

Supplementary Tables

Table S1. The negative control shows variations in the number of sensilla at specific positions. Overall, 98 to 100% of the sensilla analysed are present in control larvae. The observed variation (wt*) is due to the absence of sensilla at specific positions along the anterior-posterior axis. In the head and thorax, 99.1% of the analysed sensilla are present at all positions (2461/2484), while in the abdominal segments overall 2.87% (199/6912) of sensilla are missing. The abdominal TSOs (4.32%) show the highest variability followed by the CSGs (2.60%).

	head				
	n sensilla for 108 larvae	counted		%	
		wt	wt*	wt	wt*
BSMs	108	108	0	100.00%	0.00%
CSMs	1080	1061	19	98.24%	1.76%
CSGs	-	-	-	-	-
TSOs	108	106	2	98.15%	1.85%
	thorax				
	n sensilla for 108 larvae	counted		%	
		wt	wt*	wt	wt*
BSMs	216	215	1	99.54%	0.46%
CSMs	648	648	0	100.00%	0.00%
CSGs	324	323	1	99.69%	0.31%
TSOs	-	-	-	-	-
	abdomen				
	n sensilla for 108 larvae	counted		%	
		wt	wt*	wt	wt*
BSMs	2592	2550	42	98.38%	1.62%
CSMs	-	-	-	-	-
CSGs	1728	1683	45	97.40%	2.60%
TSOs	2592	2480	112	95.68%	4.32%

Table S2. *Tc ASH* RNAi quantification of phenotypes. The table shows the break-down of the phenotype by sensilla category and body section (head, thorax, abdomen). All larvae that showed a phenotype (i.e. missing sensilla) were included in the analysis (The table shows the break-down of the phenotype by sensilla category and body section (head, thorax, abdomen). All larvae that showed a phenotype (i.e. missing sensilla) were included in the analysis (NOF1 (n = 91) plus NOF2 (n = 172)).

	head				
	n sensilla for 263 larvae	counted		%	
		wt	phenotype	wt	phenotype
BSMs	263	1	262	0.38%	99.62%
CSMs	2630	253	2377	9.62%	90.38%
CSGs	-	-	-	-	-
TSOs	263	256	7	97.34%	2.73%
	thorax				
	n sensilla for 263 larvae	counted		%	
		wt	phenotype	wt	phenotype
BSMs	526	103	423	19.58%	80.42%
CSMs	1578	765	813	48.48%	51.52%
CSGs	789	488	301	61.85%	38.15%
TSOs	-	-	-	-	-
	abdomen				
	n sensilla for 263 larvae	counted		%	
		wt	phenotype	wt	phenotype
BSMs	6312	2570	3742	40.72%	59.28%
CSMs	-	-	-	-	-
CSGs	4208	2186	2022	51.95%	48.05%
TSOs	6312	1654	4658	26.20%	73.80%

Table S3. *Tc ato* RNAi quantification of phenotypes. The table shows the break-down of the phenotype by sensilla category and body section (head, thorax, abdomen). All larvae that showed a phenotype (i.e. missing sensilla, reduced sensilla length) were included in the analysis (NOF1 (n = 34) and NOF2 (n = 27)).

	head				
	n sensilla for 61 larvae	counted		%	
		wt	phenotype	wt	phenotype
BSMs	61	61	0	100.00%	0.00%
CSMs	610	577	33	94.59%	5.41%
CSGs	-	-	-	-	-
TSOs	61	2	59	3.28%	96.72%
	thorax				
	n sensilla for 61 larvae	counted		%	
		wt	phenotype	wt	phenotype
BSMs	122	115	7	94Z26%	5.74%
CSMs	366	354	12	96.72%	3.28%
CSGs	183	181	2	98.91%	1.09%
TSOs	-	-	-	-	-
	abdomen				
	n sensilla for 61 larvae	counted		%	
		wt	phenotype	wt	phenotype
BSMs	1464	1380	84	94.26%	5.74%
CSMs	-	-	-	-	-
CSGs	976	951	25	97.44%	2.56%
TSOs	1464	1367	97	93.37%	6.63%

Table S4. ***Tc ct*** **RNAi quantification of phenotypes.** The table shows the break-down of the phenotype by sensilla category and body section (head, thorax, abdomen). All larvae that showed a phenotype (i.e. missing sensilla, reduced sensilla length, socket only) were included in the analysis (NOF1 (n = 13) and NOF2 (n = 4)).

	head				
	n sensilla for 17 larvae	counted		%	
		wt	phenotype	wt	phenotype
**BSMs**	17	11	6	64.71%	35.29%
**CSMs**	170	71	99	41.76%	58.24%
**CSGs**	-	-	-	-	-
**TSOs**	17	1	16	5,.8%	94.12%
	**thorax**				
	**n sensilla for 17 larvae**	**counted**		**%**	
		**wt**	**phenotype**	**wt**	**phenotype**
**BSMs**	34	22	12	64.71%	35.29%
**CSMs**	102	50	52	49.02%	50.98%
**CSGs**	51	24	27	47.06%	52.94%
**TSOs**	-	-	-	-	-
	**abdomen**				
	**n sensilla for 17 larvae**	**counted**		**%**	
		**wt**	**phenotype**	**wt**	**phenotype**
**BSMs**	408	231	176	56.62%	43.14%
**CSMs**	-	-	-	-	-
**CSGs**	272	129	143	47.43%	52.57%
**TSOs**	408	46	364	11.27%	89.22%

Table S5. *Tc poxn* RNAi quantification of phenotypes. The table shows the break-down of the phenotype by sensilla category and body section (head, thorax, abdomen). All larvae that showed a phenotype (i.e. missing sensilla, duplicated sensilla) were included in the analysis (NOF1 (n = 57), NOF2 cuticles showed no phenotype).

	head				
	n sensilla for 57 larvae	counted		%	
		wt	phenotype	wt	phenotype
BSMs	57	57	0	100.00%	0.00%
CSMs	570	562	8	98.60%	1.40%
CSGs	-	-	-	-	-
TSOs	57	47	10	82.46%	17.54%
	**thorax**				
	**n sensilla for 57 larvae**	**counted**		**%**	
		**wt**	**phenotype**	**wt**	**phenotype**
BSMs	114	107	7	93.86%	6.14%
CSMs	342	249	93	72.81%	27.19%
CSGs	171	171	0	100.00%	0.00%
TSOs	-	-	-	-	-
	**abdomen**				
	**n sensilla for 57 larvae**	**counted**		**%**	
		**wt**	**phenotype**	**wt**	**phenotype**
BSMs	1368	1027	341	75.07%	24.93%
CSMs	-	-	-	-	-
CSGs	912	894	18	98.03%	1.97%
TSOs	1368	925	443	67.62%	32.38%

Table S6. Summary of RNAi injection results for both non-overlapping dsRNA fragments. RNAi phenotype categories: In the control larvae, (1) 'wt': all larvae that have 98 to 100% sensilla at the analysed positions. (2) 'Phenotype': all larvae showing recurring specific sensilla and/or other morphological phenotypes corresponding to the injected ds RNA. (3) 'Non-specific': all larvae showing sporadic morphological defects, (4) 'empty eggs': all eggs that do not develop cuticles. *Tc ASH* RNA*i*: We find similar phenotypes and range for both *Tc ASH* NOF1 and NOF2 as described in the iBeetle screen [49]. *Tc ato* RNAi: the *Tc ato* RNAi phenotype is described as 80% lethality of pupae/adults 11 days after injection. We found that ant_TSOs are missing for both *Tc ato* RNAi NOFs (penetrance of both ant_TSOs missing: NOF1, 100%; NOF2, 41%). *Tc ct* RNAi: We initially injected *Tc ct* dsRNA into female pupae. We observed a high lethality (92% for NOF1 and 84% for NOF2, 11 days after injection), as well as sterility. In the iBeetle base the results for *Tc ct* RNAi correspond to our observations, also reporting high female lethality (50% of injected pupae are dead 11 days after injection) [49]. We therefore performed embryonic RNAi and obtained larval sensilla phenotypes for both

NOFs. *Tc poxn* RNAi: In the iBeetle base several unspecific phenotypes showing less than 30% penetrance are recorded and a lethality of injected pupae or adults of 30% 11 days after injection was observed. In our injections of NOF1 (targeting the CDS), the lethality falls into a similar range (30% of pupae or adults died 11 days after injection). In contrast to the iBeetle screen results [49], we found a specific sensilla duplication phenotype. However, we did not observe a phenotype for NOF2 (targeting a different part of the CDS), despite repeating the injections.

	**negative control pRNAi**					**negative control eRNAi**				
	wt	phenotype	non-specific	empty eggs	total	wt	phenotype	non-specific	empty eggs	total
∑	86	0	52	66	204	22	0	4	0	4
%	42.16%	0.00%	25.49%	32.35%		84.62%	0.00%	15.38%	0.00%	
	***Tc ASH* NOF1**					***Tc ASH* NOF2**				
	wt	phenotype	non-specific	empty eggs	total	wt	phenotype	non-specific	empty eggs	total
∑	0	91	5	45	141	0	172	20	54	246
%	0.00%	64.54%	3.55%	31.91%		0.00%	69.92%	8.13%	21.95%	
	***Tc ato* NOF1**					***Tc ato* NOF2**				
	wt	phenotype	non-specific	empty eggs	total	wt	phenotype	non-specific	empty eggs	total
∑	0	34	17	60	111	25	27	6	50	108
%	0.00%	30.63%	15.32%	54.05%		23.15%	25.00%	5.56%	46.30%	
	***Tc ct* NOF1**					***Tc ct* NOF2**				
	wt	phenotype	non-specific	empty eggs	total	wt	phenotype	non-specific	empty eggs	total
∑	1	13	1	-	15	0	4	7	-	11
%	6.67%	86.67%	6.67%	-		0.00%	36.36%	63.64%	-	
	***Tc poxn* NOF1**					***Tc poxn* NOF2**				
	wt	phenotype	non-specific	empty eggs	total	wt	phenotype	non-specific	empty eggs	total
∑	4	57	14	36	111	63	0	22	42	
%	3.60%	51.35%	12.61%	32.43%		49.61%	0.00%	17.32%	33.07%	

Table S7. List of analysed Atonal family proteins. *D. melanogaster* sequences and other insect and vertebrate orthologues were obtained from flybase. Orthologues were verified by BLAST search on NCBI.

**Organism**	**NCBI ID**	**Description**	**(predicted) gene**
Aedes aegypti	EAT43977.1	AAEL004648-PA	*ato*
Aedes aegypti	EAT47214.1	AAEL001637-PA	*cato*

Anopheles gambiae	A0A1S4GWB7	BHLH domain-containing protein	*cato*
Anopheles gambiae str. PEST	XP_317677.3	AGAP007824-PA	*amos*
Anopheles gambiae str. PEST	XP_321345.3	AGAP001741-PA	*ato*
Apis mellifera	XP_006569960.2	uncharacterized protein LOC102656766	*amos*
Drosophila melanogaster	NP_477344.1	cousin of atonal	*cato*
Drosophila melanogaster	NP_477446.1	absent MD neurons and olfactory sensilla	*amos*
Drosophila melanogaster	NP_731223.1	atonal	*ato*
Musca domestica	XP_005184021.1	PREDICTED: protein atonal	*ato*
Musca domestica	XP_005192212.1	PREDICTED: basic helix-loop-helix transcription factor amos	*amos*
Nasonia vitripennis	XP_001602174.1	basic helix-loop-helix transcription factor amos**	*ato*
Solenopsis invicta	XP_011167744.1	protein atonal	*ato*
Strigamia maritima	T1INY9*	BHLH domain-containing protein	*amos*
Tribolium castaneum	EEZ97732.1[(1)]	atonal	*ato*
Tribolium castaneum	EFA00331.1[(2)]	Basic helix-loop-helix transcription factor amos-like Protein	*amos*
Tribolium castaneum	KYB28964.1[(3)]	Protein atonal-like Protein	*cato*

Danio rerio	AAB82272.1	atonal homologue-1	*atoH1*
Danio rerio	NP_571707.1	atonal homologue 7	*atoH7*
Musca domestica	NP_031526.1	atonal homologue 1	*atoH1*
Musca domestica	NP_001351577.1	atonal homologue 7 isoform 2	*atoH7*

*Uniprot ID; ** XP_001602174.1 is predicted as *amos* on NCBI but was listed as an *ato* orthologue on flybase; (1) same as TC011336, (2) same as TC003170, (3) same as TC032304

Table S8. Primer sequences.

Gene name	Primer	Primer sequence 5' → 3'
Tc ASH	FW	TCACCCAACCCACCAAAAC
	RV	TCACCCAACCCACCAAAAC
Tc ato	FW	CGGCTACCTCTACATGCTCA
	RV	TTGGAGCGTCTCGTACTTG
Tc ct	FW	AAATCAACAGCCTCCACGCC
	RV	TTGTACTGACTGGCGACGAG
Tc poxn	FW	CGGAATGTTCGCTTGGGAAA
	RV	TCCGCATCGTGTGTGAGAAA
Tc amos	FW	GATGGAAGAACAACAATG
	RV	TGCACTTACTGAGATACAC
Tc cato	FW	GAACTCCGAGCACCAATATC
	RV	GTGCGAGATTCGTTCCATTT
Tc tap	FW	CCACGACCTCTCCTACGAAG
	RV	TCCTTGATCGACAAACCCGT
Tc pros	FW	Biffar and Stollewerk, 2014
	RV	

Tc ase	FW	Biffar and Stollewerk, 2014
	RV	

Table S9. Double-stranded RNA information. TC numbers are listed for each gene, as well as the corresponding number from the iBeetle screen (iB number) for each fragment [49], including the concentration used for each fragment.

**gene name**	**ID**	**TC number**	**concentratio n**
Tc ASH NOF1	iB_04489	TC008433	1µg/µl
Tc ASH NOF2	iB_04489-2		1µg/µl
Tc ato NOF1	iB_09565	TC011336	1µg/µl
Tc ato NOF2	iB_09565-2		1µg/µl
Tc ct NOF1	iB_06353	TC015699	3µg/µl
Tc ct NOF2	iB_06353-2		3µg/µl
Tc poxn NOF1	iB_10412	TC034812	1µg/µl
Tc poxn NOF2	iB_10412-2		1µg/µl
Tc cato NOF1	XX-90422-1*	TC032304	1µg/µl
Tc cato NOF2	XX-90422-2*		1µg/µl
Tc tap NOF1	iB_02207	TC033042	1µg/µl
Tc tap NOF2	iB_02207-2		1µg/µl
Tc amos NOF1	iB_06876	TC003170	1µg/µl
Tc amos NOF2	iB_06876-2		1µg/µl

* Note that this gene was not analysed in the iBeetle screen [49].
